# Influence of oropharyngeal therapy with mother’s own milk on the microbiome and metabolome of very preterm infants: a pilot study

**DOI:** 10.3389/fnut.2025.1647379

**Published:** 2025-08-01

**Authors:** Wenlong Xiu, Changyi Yang, Xiaojun Lin, Baoquan Zhang, Rong Chen

**Affiliations:** ^1^College of Clinical Medicine for Obstetrics & Gynecology and Pediatrics, Fujian Medical University, Fuzhou, China; ^2^Department of Neonatology, Fujian Maternity and Child Health Hospital, Fuzhou, China

**Keywords:** preterm infants, breast milk, oral microbiota, intestinal microbiota, metabolome

## Abstract

**Background:**

Oropharyngeal therapy with mother’s own milk (OPT-MOM) may serve as a promising therapeutic approach to elicit immunoprotective and anti-inflammatory benefits for preterm infants.

**Objectives:**

This prospective pilot study aims to investigate whether OPT-MOM alters the oral microbiota, gut microbiota and metabolic profiles in very preterm infants.

**Methods:**

The eligible infants were divided into two groups: the OPT-MOM group and the control group. The OPT-MOM group received oropharyngeal administration with mother’s own milk every 3 h, starting within the first 48 h after birth and lasted for 14 days. Salivary samples and fecal samples from both groups were collected to detect microbes using 16S rRNA gene sequencing, while fecal metabolomics was measured by untargeted liquid chromatograph-mass spectrometer.

**Results:**

A total of 26 very preterm infants were enrolled in the study, with 13 assigned to each group. Our study identified distinct oral and intestinal microbiome profiles in OPT-MOM group compared to the control group. Briefly, the relative abundance of the Escherichia-Shigella and Enterobacter genera was significantly reduced in the oral cavity of preterm infants in the OPT-MOM group, while the abundance of the Rothia genus increased markedly. After 14 days of intervention, the gut microbiota of preterm infants in the OPT-MOM group exhibited a significant decrease in the abundance of the Proteobacteria phylum and a concomitant increase in the abundance of the Firmicutes phylum, which emerged as the dominant phylum. Additionally, the OPT-MOM group showed a significant increase in the relative abundance of Streptococcus and Staphylococcus genus, while a significant decrease in Enterococcus and Enterobacter genus abundance was observed in the gut microbiota. The predominant bacteria in the oral microbiota of preterm infants are highly similar to those in the intestinal microbiota. Metabolomic profiling identified that the OPT-MOM group demonstrated significantly higher levels of multiple potentially beneficial metabolites, including N-acetylneuraminic acid, myristoylcarnitine, lauroylcarnitine, acetylcarnitine, and 2,4-dihydroxybutanoic acid.

**Conclusion:**

Administration of OPT-MOM could promote the establishment of favorable microbial communities in both oral and intestinal ecosystems of preterm infants, potentially facilitating the production of metabolites that are crucial for infant health.

## 1 Introduction

Very preterm infants (VPIs), defined as gestational age less than 32 weeks, have high rates of mortality and poor outcome, commonly resulting from morbidities such as sepsis, necrotizing enterocolitis (NEC) and neurodevelopmental impairments (1–4). Breastfeeding plays an prominent role in caring of preterm infants and have been linked with improved outcomes and significant decreased incidences of prematurity-specific morbidities including LOS (late-onset sepsis), NEC and feeding intolerance compared with formula-fed cohorts (5–8). Breast milk, particularly colostrum, not only provides the best nutrition for preterm infants, but also contains substantial levels of immunoactive factors including growth factors, immunoglobulin A (IgA), lactoferrin, cytokines and lysozymes (9, 10). Besides, breast milk is also rich in bacterial communities and prebiotics such as oligosaccharides (HMOs), which promote the growth of beneficial microbes, thus influencing the colonization of bacteria in early infancy (11, 12). These immunomodulatory factors may interact with the oropharyngeal-associated lymphoid tissue via oropharyngeal contact, thereby providing antimicrobial, anti-inflammatory, antioxidant, and immunomodulatory functions. Additionally, these interactions can improve the diversity and balance of the intestinal microbiota and promote intestinal maturation (13).

However, VPIs are unable to feed orally in the first days after birth due to immaturity of sucking reflex, poor coordination between sucking and swallowing and clinical instability. They usually have to be fed through a nasogastric tube until oral feedings are introduced several weeks post-birth, which precludes the immunoprotective benefits of breast milk provided through oropharyngeal contact. In 2009, Rodriguez first proposed oropharyngeal administration of colostrum (OAC), which involves placing a small volume of colostrum into the buccal cavity of very low birth weight (VLBW) preterm infants during the first days after birth, as an alternative to oropharyngeal contact with colostrum, allowing preterm infants to reap the benefits of oropharyngeal contact with breast milk before oral feeding is established (14). Accumulating evidence has suggested that OAC is a feasible and safe intervention for VPIs and could potentially promote gastrointestinal immune function, reduce the risk of LOS, NEC, ventilator-associated pneumonia (VAP), shorten the time to achieve full enteral feeding and length of hospital stay, enhance the rate of weight gain, thereby improving survival and long-term outcome (15–18). The milk expressed by mothers of VPIs is different from milk expressed at term and may be uniquely suited to the host defense requirements of the preterm infants (11, 19, 20). Although the highest concentrations of many protective biofactors are found in colostrum which is conventionally defined as breast milk secreted with 5 days after delivery, the levels remain high in preterm milk for several weeks after delivery (21, 22). More recently, some units have applied oropharyngeal therapy with mother’s own milk (OPT-MOM) in VPIs for more than 5 days, which could be extended until 32 weeks corrected gestational age or they could receive oral feed (23). In our pilot study, VPIs who received OPT-MOM for 10 days had lower incidence of NEC, LOS and severe intraventricular hemorrhage (IVH), compared with control groups (13).

Nevertheless, our understanding on how OAC or OPT-MOM exerts protective effects on preterm infants remains limited. The effect of OAC or OPT-MOM on the oral and intestinal microbiota may play a pivotal role in the protective mechanism. Previous studies have reported that OAC or OPT-MOM can influence the colonization of the oral microbiota in preterm infants (24–27), yet evidence regarding its impact on gut microbiota remains lacking. In addition to the direct effects of modified microbial ecosystems, microbial metabolites may serve as signals to mediate various intracellular signaling pathways, contributing significantly to the development of the immune system in early life (28). However, the impact of OAC or OPT-MOM on intestinal microbial metabolites remains unclear.

We hypothesize that OPT-MOM may influence the colonization of oral microbiota and gut microbiota, potentially leading to subsequent alterations in intestinal metabolic profiles, thereby conferring benefits to preterm infants. Therefore, we conducted a study to investigate the impact of OPT-MOM on the microbiota and metabolites in VPIs by examining dynamic changes in oral, gut microbiota, intestinal metabolites using 16S rRNA sequencing and untargeted liquid chromatograph-mass spectrometer.

## 2 Materials and methods

### 2.1 Study design and participants

This study is a prospective pilot trial conducted across two level III Neonatal Intensive Care Units (NICUs) located at distinct campuses of Fujian Provincial Maternity and Child Health Hospital, China. The study was reviewed and approved by the Ethics Committee of Fujian Provincial Maternity and Child Health Hospital (No. 2021KLRD09028) and registered at the Chinese Clinical Trial Registry (Registration ID: ChiCTR 1,900,023,697).

All inborn infants born at less than 32 weeks’ gestation and admitted to NICUs in Fujian Provincial Maternity and Child Health Hospital between 1 March 2023 and 30 August 2023 were eligible for this study. The exclusion criteria were as follows: (1) mothers prohibited from breastfeeding due to active tuberculosis or AIDS or unable to provide mother’s own milk for various reasons; (2) birth complicated with severe gastrointestinal malformations or fatal congenital chromosomal abnormalities; (3) mothers with chorioamnionitis; (4) inability to collect samples during hospitalization due to discharge against medical advice, death or other reasons; (5) infants on antibiotics for more than 7 days; (6) refusal of guardians to participate in the study.

Preterm infants were screened for eligibility upon admission to NICU. Informed consent was obtained from the guardians before participation in the study. OAC is recommend to reduce feeding intolerance in clinical guidelines for the diagnosis and treatment of feeding intolerance in China (29). Therefore, it was not feasible to randomize eligible infants into OPT-MOM or control group due to ethical considerations. Some parents were unwilling or unable to provide mother’s own milk during the initial postnatal period, thus we allocated the VIPs into two groups based on their parents’ willingness and choices. All enrolled participants received standardized care in accordance with the unified NICU protocols, which were rigorously implemented by the medical staff and maintained complete consistency across both study campuses. Both the control group and the OPT-MOM group received enteral nutrition via a nasogastric tube throughout the 14 days oropharyngeal colostrum administration period. Maternal breast milk was the primary source of enteral nutrition for both groups. When maternal milk was insufficient, donor milk or formula was used for supplementation. Feeding volumes were gradually increased for all infants based on their clinical tolerance, with milk administered as intermittent bolus feeds. Both groups were administered parenteral nutrition and caffeine intravenously. None of the infants in either group were administered any oral medications, except for one infant who received ibuprofen for patent ductus arteriosus closure. Maternal and infant data were collected until discharge or death.

Necrotizing enterocolitis was defined as neonates with stage ≥ II NEC according to the Bell criteria (30, 31). Late-onset sepsis (LOS), including proven and clinical sepsis, was defined as sepsis occurring at > 72 h of life (32). Retinopathy of prematurity (ROP) was diagnosed according to the International Classification of Retinopathy of Prematurity (33). Bronchopulmonary dysplasia (BPD) was defined as ventilation or oxygen dependency at 36 weeks’ corrected age or at discharge, transfer, or death before 36 weeks (34). Severe intraventricular hemorrhage (IVH) (diagnosis and classification by ultrasound as grade 3 or 4) (35).

### 2.2 Oropharyngeal administration procedure

Mothers of preterm infants were instructed to milk frequently with hygienic hands to ensure consistent breast milk production and supply. In the OPT-MOM group, the mother’s milk was collected in a pre-labeled sterile disposable sealed cups. The mother’s milk was sent to NICU by cold-chain for acceptance and registration within 4 h after collection. The OPT-MOM intervention was initiated following a standardized protocol within 48 hours after birth and maintained for a duration of 14 consecutive days. Following warming in a water bath at 37°C, 0.4 ml of breast milk was aspirated into a sterile syringe by the bedside nurse. Subsequently, 0.2 ml of breast milk was dripped into each side of the infant’s oropharyngeal cavity and applied evenly on the cheeks, palate, lingual surface, gingiva and lips for a minimum duration of 2 min every 3 h, using a sterile silicone fingerstall. In the control group, the bedside nurse implemented identical administration protocols as those utilized in the OPT-MOM group, with the exception that sterile normal saline was substituted for breast milk.

### 2.3 Sample collection

Salivary samples and stool samples were collected from participants from both groups at predefined intervals: baseline (T0, the first day of life), the 10th day of life (T1), and the 20th day of life (T2). The first saliva sampling was performed prior to oropharyngeal intervention initiation. All specimens were obtained using aseptic technique. After collection, samples were immediately frozen in liquid nitrogen and transferred to −80°C cryogenic storage within 30 min of collection until they were sent for further analysis.

### 2.4 Microbial 16S rRNA gene sequence analysis

DNA from samples was extracted using the CTAB method according to manufacturer’s instructions. The V3–V4 region of bacterial 16S rRNA gene was amplified via Polymerase Chain Reaction (PCR) using the forward primers 341F (5′-CCTACGGGNGGCWGCAG-3′) and the reverse primer 806R (5′-GACTACHVGGGTATCTAATCC-3′). The PCR products were purified using AMPure XT beads (Beckman Coulter Genomics, Danvers, MA, United States) and quantified by Qubit (Invitrogen, United States). Samples were sequenced on an Illumina NovaSeq platform.

Bioinformatics analysis was primarily conducted using QIIME2 and R (version 3.4.4). The workflow included data demultiplexing, data assembly and filtering, DADA2 dereplication, diversity analysis, taxonomic annotation, differential analysis, and advanced statistical analyses. The taxonomy was assigned using ASVs (Amplicon Sequencing Variant). Alpha diversity was measured by the indices including Chao1, Shannon, and Simpson indices, which evaluate the richness and evenness of the microbiota across different sample groups. Beta diversity was assessed using principal coordinates analysis (PCoA) based on the weighted UniFrac distance matrix, in order to explore the differences in the composition and structure of the global microbiota among groups. Taxonomic annotation were performed using the SILVA and NT-16S databases. The relative abundances of taxa were determined from the ASV abundance table, applying a confidence threshold of 0.7. Differential abundance analysis of dominant bacteria was conducted using the Mann-Whitney U test, with a significance threshold of *p* < 0.05. Linear discriminant analysis Effect Size (LEfSe) was used to identify the differentially abundant taxa responsible for the differences between participant groups. The threshold for the logarithmic Linear Discriminant Analysis (LDA) score was set to 3.0 to identify discriminative features. Finally, BugBase was applied to predict bacterial phenotypes, in order to infer potential functional roles of the microbiota.

### 2.5 Fecal metabolome analysis by liquid chromatography–mass spectrometry

Metabolites were extracted from fecal samples using an extraction solution containing isotopically labeled internal standards. Untargeted liquid chromatography-tandem mass spectrometry (LC-MS) analysis was applied to detect metabolites. Chromatographic separation of target compounds was achieved using a Waters ACQUITY UPLC BEH Amide column on a Vanquish ultra-high-performance liquid chromatography system. Primary and secondary mass spectrometry data were acquired using an Orbitrap Exploris 120 mass spectrometer, controlled by Xcalibur software (version 4.4, Thermo).

The raw data were converted into mzXML format using ProteoWizard software, and metabolite identification was performed using a custom-developed R package. The database employed for identification was BiotreeDB (V3.0). The multivariate and univariate statistical analyses of the obtained data were performed using the R software package. The multivariate statistical analysis was performed by Orthogonal Projections to Latent Structures-Discriminant Analysis (OPLS-DA). Differential metabolites were identified based on the *P*-value from Student’s *t*-test and the Variable Importance in Projection (VIP) score from the first principal component of the OPLS-DA model. Metabolites with VIP > 1 and *P* < 0.05 were considered differentially expressed. Metabolic pathways associated with the differentially expressed metabolites were annotated, classified, and enriched using the Kyoto Encyclopedia of Genes and Genomes (KEGG) database. Comprehensive pathway analysis was performed using enrichment analysis and topological analysis to identify key pathways most strongly correlated with the observed metabolic differences. The results were visualized using a self-developed R package.

### 2.6 Correlation analysis

A combined analysis was constructed based on Spearman correlation analysis to investigate the complex relationships between oral microbiome and gut microbiome, as well as between the gut microbiome and gut metabolome. Briefly, the correlation coefficients and statistical significance were calculated for the differentially abundant metabolites and microbes previously identified.

### 2.7 Statistical analysis

The clinical data of Control group and OPT-MOM group were analyzed by using SPSS 26.0 Statistics for Windows. Data are presented as mean ± standard deviation, median (interquartile range), or counts (percentages), as appropriate. The normality and homogeneity of variance of the measurement data were assessed using the Kolmogorov–Smirnov test and Levene’s test, respectively. For continuous variables, Student’s *t*-test was employed for parametric data, while the Mann–Whitney U test was used for non-parametric data. Categorical variables were analyzed using the chi-squared test or Fisher’s exact test, as appropriate. A *P*-value of less than 0.05 was considered statistically significant.

For microbiome and metabolome data analysis, specific matched statistics methods were used, as mentioned above.

## 3 Results

### 3.1 Demographics and clinical characteristics

**FIGURE 1:**
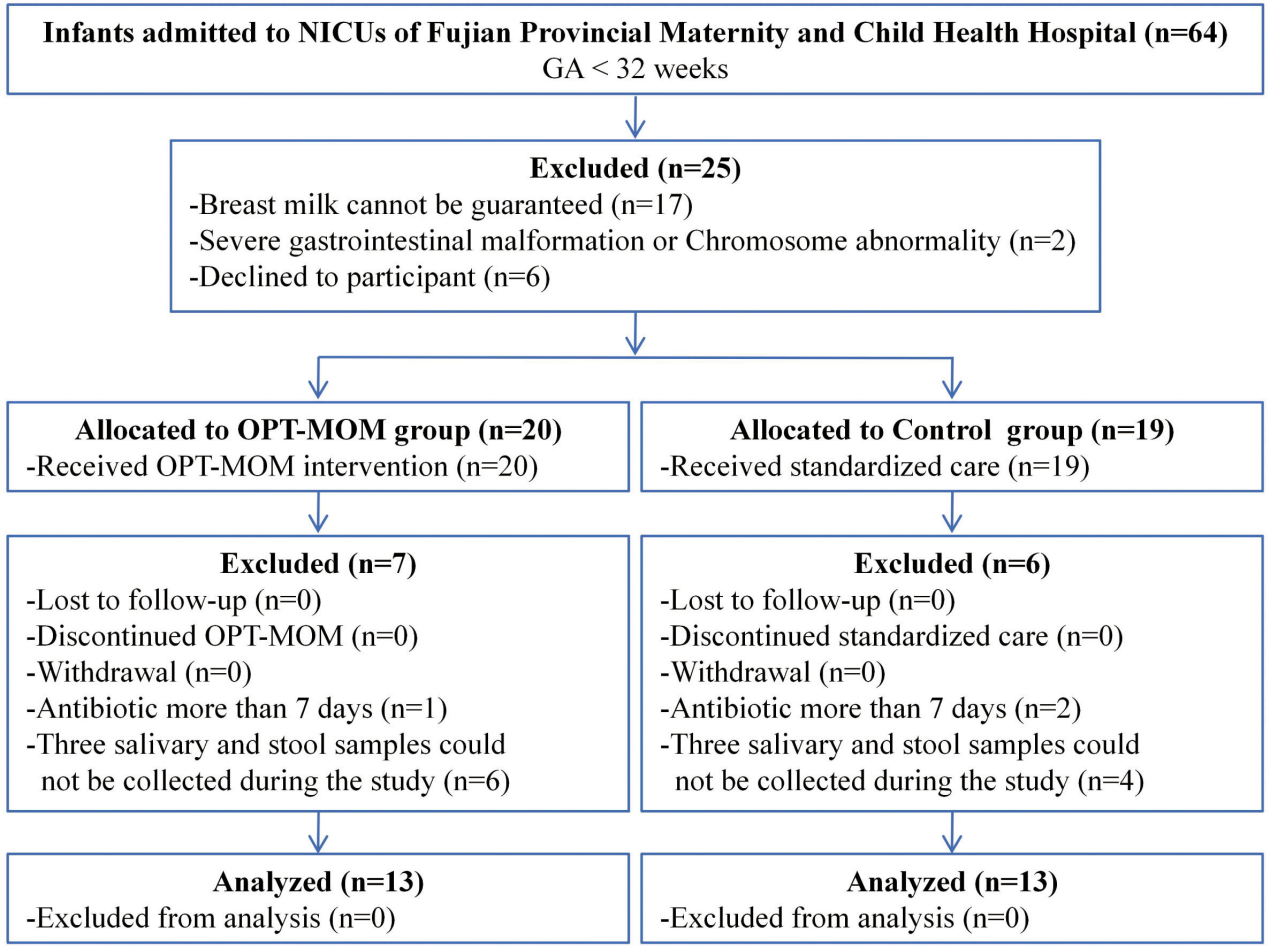
Participant flow diagram.

From 1 March 2023 to 30 August 2023, a total of 26 very preterm infants were enrolled in the study, comprising 13 in the OPT-MOM group and 13 in the control group (Figure 1). A total of 78 samples (three samples in each infant) were prospectively collected from the study population for subsequent analyses.

Maternal age was significantly higher in the OPT-MOM group compared to the controls. No other statistically significant differences in baseline characteristics were observed between the two groups (Table 1).

**TABLE 1:** Baseline characteristics between groups.

Clinical characteristics	OPT-MOM (*n* = 13)	Control (*n* = 13)	t/U/χ^2^-value	*P*-value
**Infant characteristics**				
Sex (male), *n* (%)	7 (53.85)	8 (61.54)	-^a^	> 0.9999
Gestational age (weeks), median (IQR)	30.43 (30.29, 31.29)	30.57 (29.36, 30.71)	63.50	0.29
Birthweight (g), median (IQR)	1,520 (1,215, 1,568)	1,330 (1,243, 1,475)	60.00	0.22
Small for gestational age infant, *n* (%)	0	0	–	–
Cesarean section, n (%)	7 (53.85)	10 (76.92)	-^a^	0.41
Apgar score at 5 min, median (IQR)	10 (10, 10)	10 (10, 10)	78.00	> 0.9999
**Maternal characteristics**				
Maternal age	35 (32.5, 35)	32 (27, 32,5)	35.00	0.01
Maternal body mass index	26.56 (21.55, 29.10)	25.4 (24.55, 28.62)	80.00	0.83
Multiple pregnancies	5 (38.46)	3 (23.08)	-^a^	0.67
Cervical incompetence	0	0	–	–
Placental abruption	3 (23.08)	5 (38.46)	-^a^	0.67
Premature rupture of membranes, *n* (%)	2 (15.38)	2 (15.38)	-^a^	> 0.9999
Intrapartum maternal antibiotic therapy, *n* (%)	2 (15.38)	5 (38.46)	-^a^	0.38
Gestational diabetes mellitus	5 (38.46)	5 (38.46)	-^a^	> 0.9999
Gestational hypertension	2 (15.38)	5 (38.46)	-^a^	0.67
**Medication**				
Parenteral nutrition	13	13	–	–
Caffeine (intravenous)	13	13	–	–
Ibuprofen (oral)	1 (7.69)	0	-^a^	> 0.9999

-*^a^*Fisher’s exact test: χ2 value cannot be calculated. OPT-MOM, oropharyngeal therapy with mother’s own milk group; Control, control group; IQR, interquartile range.

The clinical outcomes of both groups are presented in Table 2. No statistically significant differences were observed between group in the incidence of NEC, late-onset Sepsis, BPD and ROP (all *p* > 0.05). And there were no significant differences between the two groups in the rate of mechanical ventilation, duration of respiratory support, age at achieving full enteral feeding, age at achieving full oral feeding, weight gain velocity, or length of hospital stay (all *p* > 0.05).

**TABLE 2:** Clinical outcomes between groups.

Morbidities and characteristics	OPT-MOM (*n* = 13)	Control (*n* = 13)	t/U/χ^2^-value	*P*-value
Late onset sepsis, *n* (%)	0	1 (7.69)	-^a^	> 0.9999
Necrotizing enterocolitis (Bell stage 2 or 3), *n* (%)	1 (7.69)	2 (15.38)	-^a^	> 0.9999
Bronchopulmonary dysplasia, *n* (%)	3 (23.08)	4 (30.77)	-^a^	> 0.9999
Intraventricular hemorrhage (grade 3 or 4), *n* (%)	0	0	–	–
Retinopathy of prematurity, *n* (%)	0	3 (23.08)	-^a^	0.22
Invasive mechanical ventilation, *n* (%)	2 (15.38)	2 (15.38)	-^a^	> 0.9999
Duration of respiratory support (d), median (IQR)	5 (5, 6.5)	8 (5.5, 15)	54.50	0.12
Age of achieving full enteral feeding (d), median (IQR)	30 (25.5, 34.5)	24 (20, 33)	58.50	0.19
Age of achieving full oral feeding (d), median (IQR)	31 (26, 40)	33 (29.5, 45)	65.00	0.33
Rate of weight gain (g/kg.d), mean ± SD	14.83 ± 5.123	16.62 ± 3.608	1.03	0.32
Length of hospitalization (d), mean ± SD	46.38 ± 16.34	57.92 ± 23.39	48.00	0.06

-*^a^*Fisher’s exact test: χ2 value cannot be calculated. OPT-MOM, oropharyngeal therapy with mother’s own milk group; Control, control group; IQR, interquartile range; SD, standard deviation.

### 3.2 Oral bacteria

The numbers of shared and distinct ASVs of oral bacteria between groups were visualized in a Venn diagram (Supplementary Figure 1A), while the counts of shared and unique ASVs in each group at different time points were illustrated in Supplementary Figure 1B.

**FIGURE 2:**
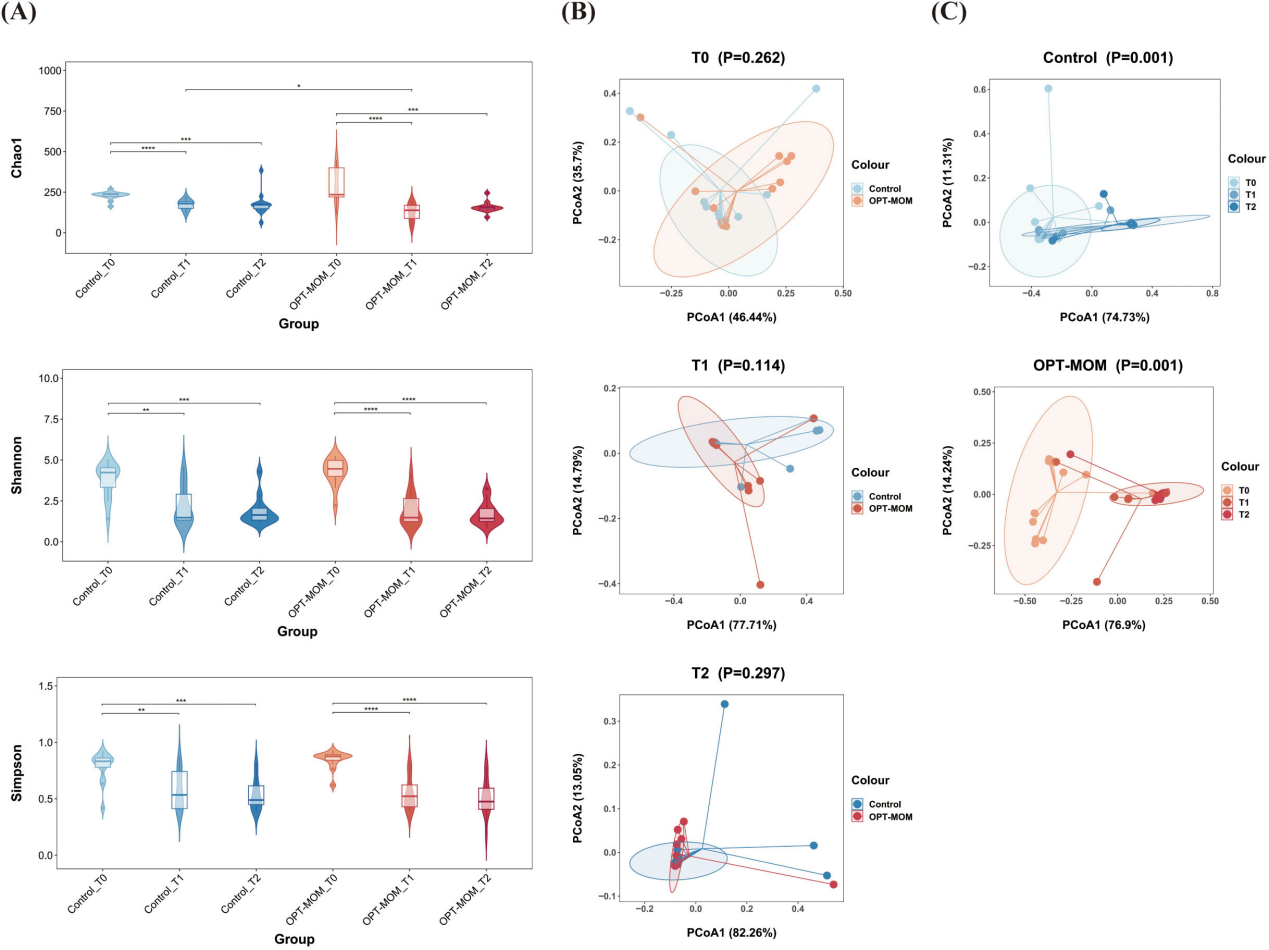
Alpha and Beta diversities of oral microbiota in control group and OPT-MOM group over time. **(A)** Alpha diversity of two groups at different times. **(B)** Beta diversity comparison between the two groups at different times. **(C)** Beta diversity of control group and OPT-MOM group over time. Control, control group; OPT-MOM, oropharyngeal therapy with mother’s own milk group. T0, the first day of life; T1, the 10th day of life; T2, the 20th day of life. *< 0.05, **< 0.01, ***< 0.001, ****< 0.0001.

The alpha diversity and beta diversity indices of oral bacteria in two groups at different time points can be seen in Figure 1. A significant reduction in the Chao1 index was observed in the OPT-MOM group compared to the control group on the 10th day of life (T1). However, no statistically significant differences were detected in the Chao1 index between the two groups on the first day of life (T0) and the 20th day of life (T2). There is no significant difference between the two groups in the Shannon and Simpson indices at any of the three time points. When assessing the impact of postnatal age on the oral bacteria, we observed that the three indices of alpha diversity were significantly reduced at T1 and T2 compared to T0, in both the OPT-MOM and control groups (Figure 2A). Regarding beta diversity, principal coordinates analysis (PCoA) revealed no significant differences in oral microbial composition between the two groups at any of the three time points (Figure 2B). However, when time was considered as a variable, the oral microbial structure differed significantly over time in both the OPT-MOM and control groups (Figure 2C).

**FIGURE 3:**
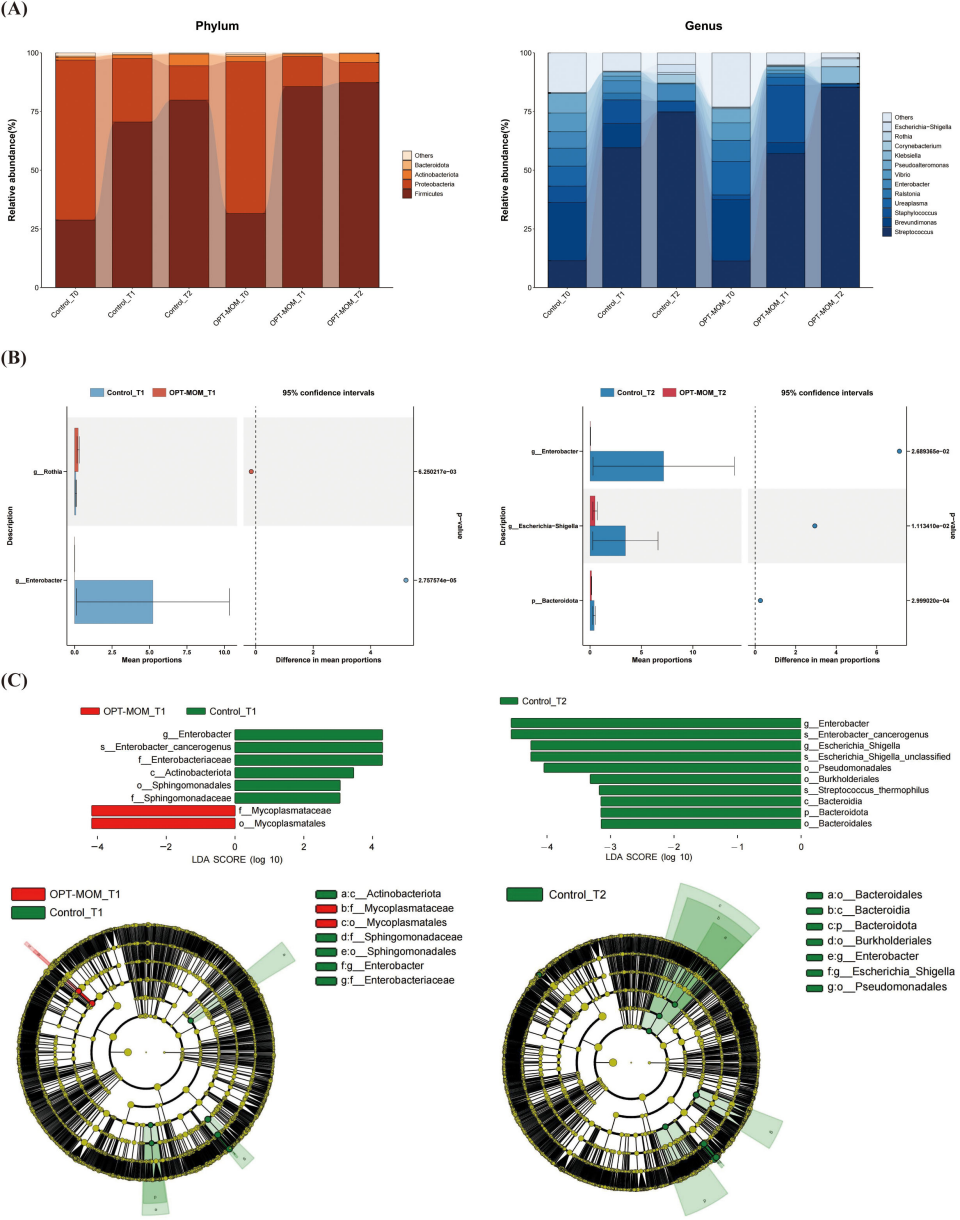
Comparison of the relative abundance of the oral microbiota between the control and OPT-MOM groups. **(A)** Relative abundance of top 4 phylum and the top 12 genus of two groups at different times. **(B)** Differentially dominant species comparison between the two groups at different times. **(C)** Differentially abundant microbial clades between the two groups at different times. Control, control group; OPT-MOM, oropharyngeal therapy with mother’s own milk group. T0, the first day of life; T1, the 10th day of life; T2, the 20th day of life.

The oral flora structure and relative bacterial abundances in the saliva samples were shown at the phylum level and genus level (Figure 3A). At the phylum level, the oral microbial communities in both OPT-MOM and control groups exhibited consistent temporal dynamics in their predominant bacterial composition. At baseline (T0), the most predominant bacterial phylum in both groups was Proteobacteria, followed by Firmicutes (2nd) and Actinobacteria (3rd) in descending order of relative abundance. At subsequent time points (T1 and T2), the phylum Firmicutes emerged as the most predominant, followed by Proteobacteria (2nd) and Actinobacteria (3rd) in both groups. At the genus level, the relative abundance of the most predominant genera in the control and OPT-MOM groups exhibited distinct patterns over time. At baseline (T0), the top three genera in the control group were *Brevundimonas*, *Streptococcus*, and *Ureaplasma*, while in the OPT-MOM group, they were *Brevundimonas*, *Ureaplasma*, and *Streptococcus*. At T1, the most abundant genera in the control group shifted to *Streptococcus*, *Brevundimonas*, and *Staphylococcus*, whereas in the OPT-MOM group, they were *Streptococcus*, *Staphylococcus*, and *Brevundimonas*. By T2, the control group was dominated by *Streptococcus*, *Enterobacter*, and *Staphylococcus*, while the OPT-MOM group was characterized by *Streptococcus*, *Klebsiella*, and *Rothia*.

Bacteria that exhibited significant differences in abundance between the two groups at T1 and T2 are illustrated in Figure 3B. In comparison with the control group, at T1, the relative abundance of *Rothia* was higher in the OPT-MOM group, while the relative abundance of *Enterobacter* was lower. At T2, the relative abundance of *Bacteroidota*, *Enterobacter*, and *Escherichia-Shigella* was lower in the OPT-MOM group.

The discrepant microbial species with a significance threshold (LDA score > 3) between the two groups were shown in Figure 3C. The LEfSe analysis revealed significant differences in the relative abundance of specific microbial taxa between the OPT-MOM group and the control group at different time points (T1 and T2). At T1, the relative abundance of several taxa within the Proteobacteria phylum, including *Enterobacter* genus, *Enterobacter cancerogenus*, Enterobacteriaceae family, Sphingomonadales order, and Sphingomonadaceae family, was significantly decreased in the OPT-MOM group compared to the control group. Additionally, the Actinobacteriota class (which belongs to the Actinobacteria phylum) also exhibited a reduced relative abundance. In contrast, the OPT-MOM group demonstrated a significant increase in the relative abundance of Mycoplasmatales order and Mycoplasmataceae family, both of which are phylogenetically classified in the Firmicutes phylum. At T2, the relative abundance of the *Enterobacter* genus, *Enterobacter cancerogenus*, *Escherichia-Shigella* genus, and unclassified species within the *Escherichia-Shigella* genus, Pseudomonadales order and Burkholderiales order (all belonging to the Proteobacteria phylum) was decreased in the OPT-MOM group. Moreover, the OPT-MOM group exhibited a significant reduction in the relative abundance of *Streptococcus thermophilus* (belonging to the Firmicutes phylum), along with decreased levels of taxa affiliated with the Bacteroidetes phylum, including Bacteroidia class and Bacteroidales order.

To infer the functional characteristics of the oral microbiota in preterm infants, we used BugBase to predict bacterial phenotypes (Supplementary Figure 1C). Our analysis revealed that the oropharynx of preterm infants was predominantly colonized by aerobic bacteria and gram-negative bacteria on the first day after birth (T0). At 20 days after birth (T2), the oral microbiota was characterized by a higher abundance of facultatively aerobic bacteria and gram-positive bacteria. At T2, the oral microbiota of the OPT-MOM group exhibited a trend toward lower potential pathogenicity compared to the control group. However, this difference did not reach statistical significance (*p* = 0.10).

### 3.3 Gut bacteria

The numbers of shared and distinct ASVs of gut bacteria between groups were depicted in Supplementary Figure 2A, while the counts of shared and unique ASVs in each group at different time points were illustrated in Supplementary Figure 2B.

**FIGURE 4:**
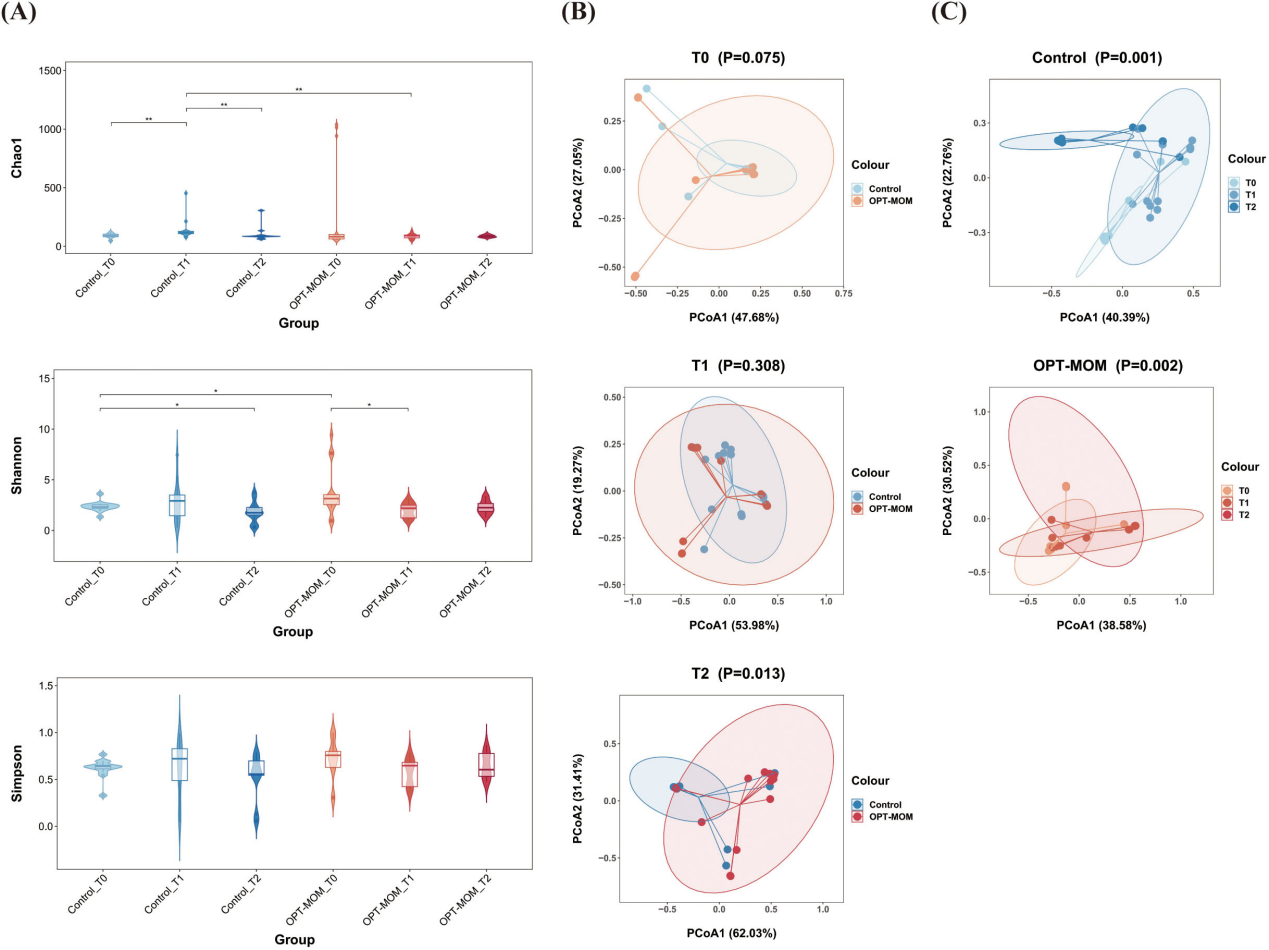
Alpha and Beta diversities of gut microbiota in control group and OPT-MOM group over time. **(A)** Alpha diversity of two groups at different times. **(B)** Beta diversity comparison between the two groups at different times. **(C)** Beta diversity of control group and OPT-MOM group over time. Control, control group; OPT-MOM, oropharyngeal therapy with mother’s own milk group. T0, the first day of life; T1, the 10th day of life; T2, the 20th day of life. *< 0.05, **< 0.01.

The alpha diversity and beta diversity indices of gut bacteria in two groups at different time points were presented in Figure 4. The Chao1 index was significantly lower in the OPT-MOM group compared to the control group at T1. In the control group, the Chao1 index increased significantly from T0 to T1 and then decreased significantly from T1 to T2. No statistically significant differences were observed in the Chao1 index across different time points within the OPT-MOM group. The OPT-MOM group exhibited a higher Shannon index than the control group at T0. In the control group, the Shannon index decreased significantly from T0 to T2. In the OPT-MOM group, the Shannon index decreased significantly from T0 to T1. No significant differences were observed in the Simpson indices between the two groups at any of the three time points. And there were also no significant differences in the Simpson indices among different time points within each group (Figure 4A). PCoA showed no difference in gut microbial composition between the two groups at T0 and T1. However, a significant difference was observed at T2 (Figure 4B). In addition, significant differences in the beta diversity of the gut microbiota were observed across the three time points in both the OPT-MOM and control groups (Figure 4C).

**FIGURE 5:**
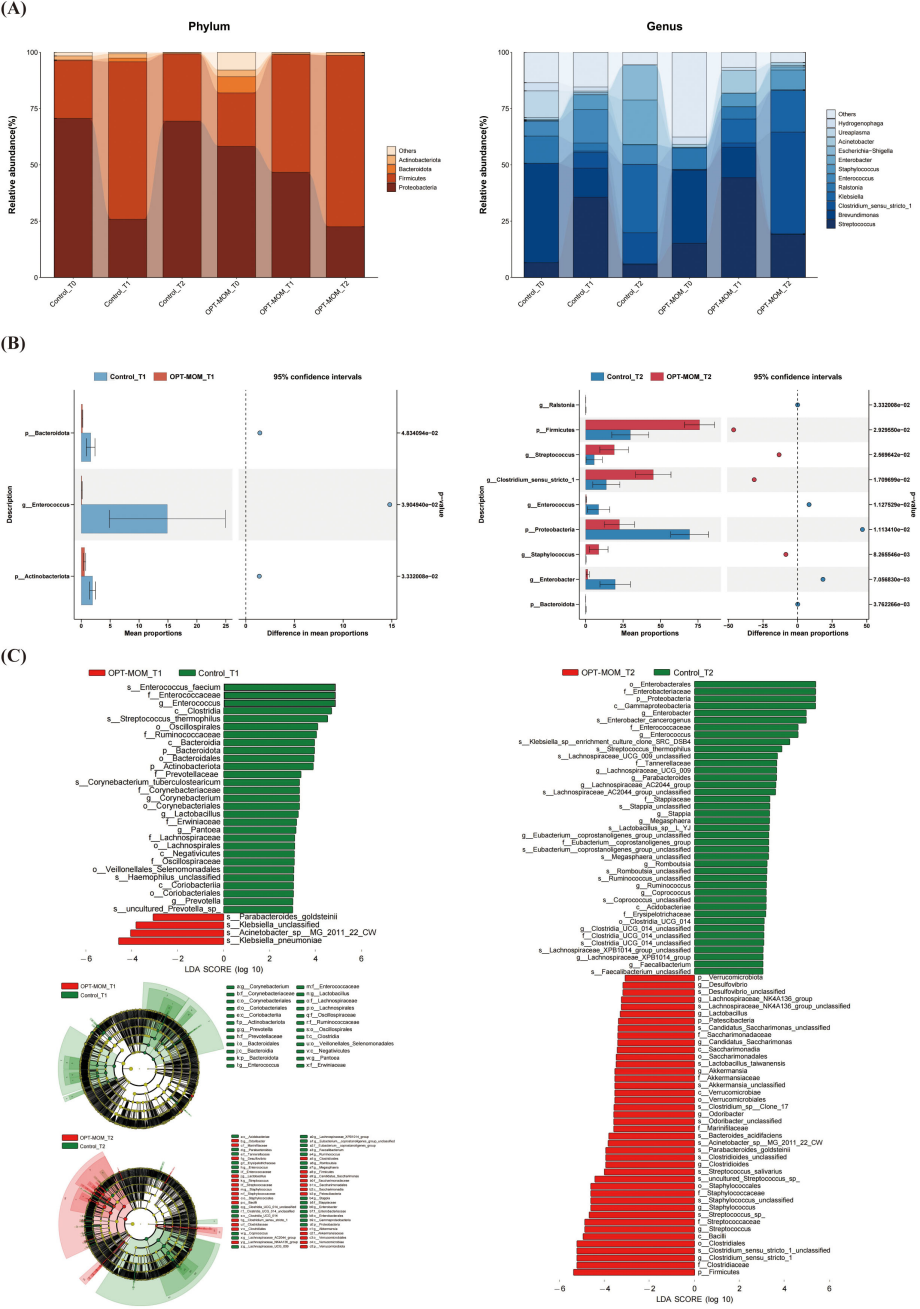
Comparison of the relative abundance of the gut microbiota between the control and OPT-MOM groups. **(A)** Relative abundance of top 4 phylum and the top 12 genus of two groups at different times. **(B)** Differentially dominant species comparison between the two groups at different times. **(C)** Differentially abundant microbial clades between the two groups at different times. Control, control group; OPT-MOM, oropharyngeal therapy with mother’s own milk group. T0, the first day of life; T1, the 10th day of life; T2, the 20th day of life.

The gut flora structure and relative bacterial abundances in the stool samples were presented at the phylum level and genus level (Figure 5A). At the phylum level, the top three phyla in the control group at baseline (T0) were Proteobacteria, Firmicutes, and Actinobacteria, while in the OPT-MOM group, they were Proteobacteria, Firmicutes, and Bacteroidota. At T1, the predominant phyla in both groups shifted to Firmicutes, Proteobacteria, and Actinobacteria. By T2, the control group exhibited a shift in the order of abundance to Proteobacteria, Firmicutes, and Actinobacteria, whereas the OPT-MOM group maintained the same top three phyla as T1. At the genus level, the top three genera in abundance for the control group at baseline (T0) were *Brevundimonas*, *Ralstonia*, and *Ureaplasma*, while for the OPT-MOM group they were *Brevundimonas*, *Streptococcus*, and *Ralstonia*. At T1, the control group exhibited a shift in the most abundant genera to *Streptococcus*, *Enterococcus*, and *Brevundimonas*, while the OPT-MOM group was characterized by *Streptococcus*, *Brevundimonas*, and *Klebsiella*. By T2, the control group was dominated by *Klebsiella*, *Enterobacter*, and *Escherichia-Shigella*, whereas the OPT-MOM group was characterized by *Clostridium* sensu stricto 1, *Streptococcus*, and *Klebsiella*.

As shown in Figure 5B, significant intergroup differences in gut bacterial abundance were observed at both T1 and T2. At T1, the OPT-MOM group exhibited significantly lower relative abundances of *Bacteroidota*, *Actinobacteriota*, and *Enterococcus* compared to the control group. By T2, the OPT-MOM group showed a substantial increase in the relative abundance of *Firmicutes*, *Streptococcus*, *Staphylococcus*, and *Clostridium* sensu stricto 1, while a significant decrease was observed in the abundance of *Proteobacteria*, *Ralstonia*, *Enterococcus*, and *Enterobacter*.

Figure 5C showed the taxonomic groups with significant differences (LDA score > 3) between the two groups. The LEfSe analysis revealed significant microbial disparities between groups, with 29 taxa showing marked depletion and 4 taxa exhibiting enrichment in the OPT-MOM group at T1 compared to the control group. The most prominent taxa with decreased relative abundance in the OPT-MOM group at T1 were Enterococcus faecium, Enterococcaceae family, and *Enterococcus* genus. The taxa that exhibited the most substantial increases in relative abundance were *Klebsiella pneumoniae*, *Acinetobacter* sp. MG_2011_22_CW, and unclassified species within the genus *Klebsiella*. However, these increases were predominantly observed in only two samples from the OPT-MOM group. At T2, the OPT-MOM group exhibited a substantial difference in microbial community structure compared to the control group, with 41 key taxonomic units showing decreased abundance and 42 showing increased abundance. Notably, the top 3 depleted taxa included the Enterobacterales order, Enterobacteriaceae family, and Proteobacteria phylum, while the top 3 enriched taxa comprised the Firmicutes phylum, Clostridiaceae family, and Clostridium sensu stricto 1 genus.

Phenotypic profiling suggested distinct shifts in the intestinal microbiota of preterm infants over time. At T0, the gut of preterm infants was predominantly colonized by aerobic bacteria and gram-negative bacteria. In contrast, at T2, the gut microbiota was characterized by a higher abundance of anaerobic bacteria and gram-positive bacteria. Notably, after 14 days of OPT-MOM intervention, the levels of Gram-negative bacteria and potentially pathogenic bacteria in the gut were significantly reduced compared to the control group (*P* = 0.01; Supplementary Figures 2C, D).

### 3.4 Gut metabolites

**FIGURE 6:**
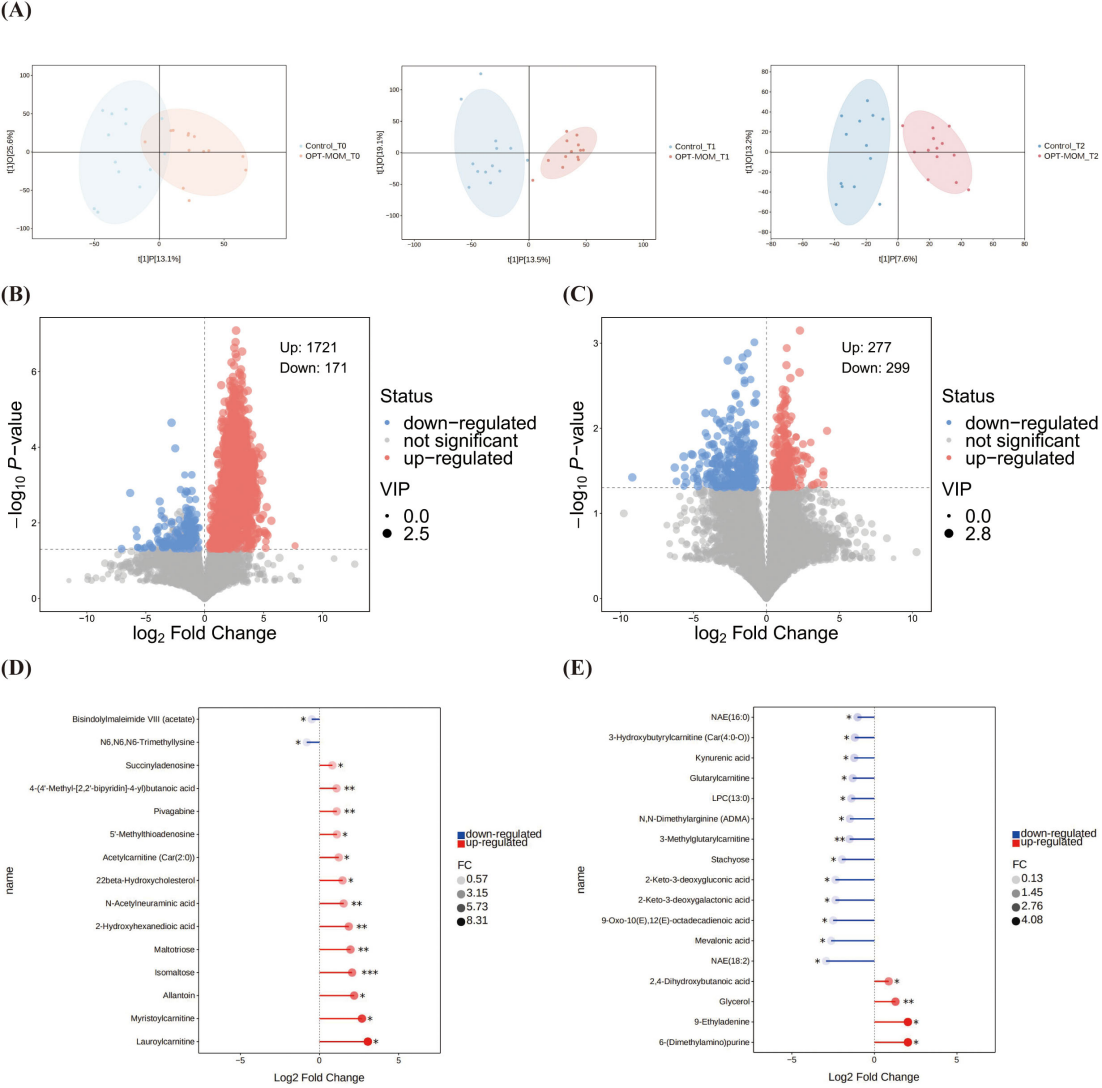
Comparison of gut metabolites between control group and OPT-MOM group at different times. **(A)** OPLS-DA analysis between groups at different times. **(B)** Volcano plots of differential metabolites between groups at T1. **(C)** Volcano plots of differential metabolites between groups at T2. **(D)** Stick plots of differential metabolites between groups at T1. **(E)** Stick plots of differential metabolites between groups at T2. Control, control group; OPT-MOM, oropharyngeal therapy with mother’s own milk group. T0, the first day of life; T1, the 10th day of life; T2, the 20th day of life. *< 0.05, **< 0.01, ***< 0.001.

Orthogonal Projections to Latent Structures-Discriminant Analysis identified significant differences in the gut metabolic profiles between the OPT-MOM group and the control group at both T2 and T3 (Figure 6A). The intestinal metabolite profiles of both groups exhibited substantial changes with increasing postnatal age (Supplementary Figure 3).

The volcano plot demonstrates the overall distribution of differential metabolites between groups (Figures 6B, C). At T1, 15 major differential metabolites were identified between the OPT-MOM and control groups. Thirteen metabolites were significantly elevated in the OPT-MOM group compared to the control group, including lauroylcarnitine, myristoylcarnitine, allantoin, isomaltose, maltotriose, 2-hydroxyhexanedioic acid, N-acetylneuraminic acid, 22beta-hydroxycholesterol, acetylcarnitine [Car(2:0)], 5’-methylthioadenosine, pivagabine, 4-(4’-methyl-[2,2’-bipyridin]-4-yl)butanoic acid, succinyladenosine, while two metabolites were markedly reduced, including N6,N6,N6-trimethyllysine and bisindolylmaleimide VIII (acetate) (Figure 6D).

At T2, 17 significantly differential metabolites were identified between the OPT-MOM and control groups. Among these, four metabolites were significantly elevated in the OPT-MOM group, including 6-(dimethylamino) purine, 9-ethyladenine, glycerol and 2,4-dihydroxybutanoic acid. And 13 metabolites were significantly reduced, encompassing NAE(18:2), mevalonic acid, 9-oxo-10(E),12(E)-octadecadienoic acid, 2-keto-3-deoxygalactonic acid, 2-keto-3-deoxygluconic acid, stachyose, 3-methylglutarylcarnitine, N,N-dimethylarginine (ADMA), LPC(13:0), glutarylcarnitine, kynurenic acid, 3-hydroxybutyrylcarnitine [Car(4:0-O)], NAE(16:0) (Figure 6E).

**FIGURE 7:**
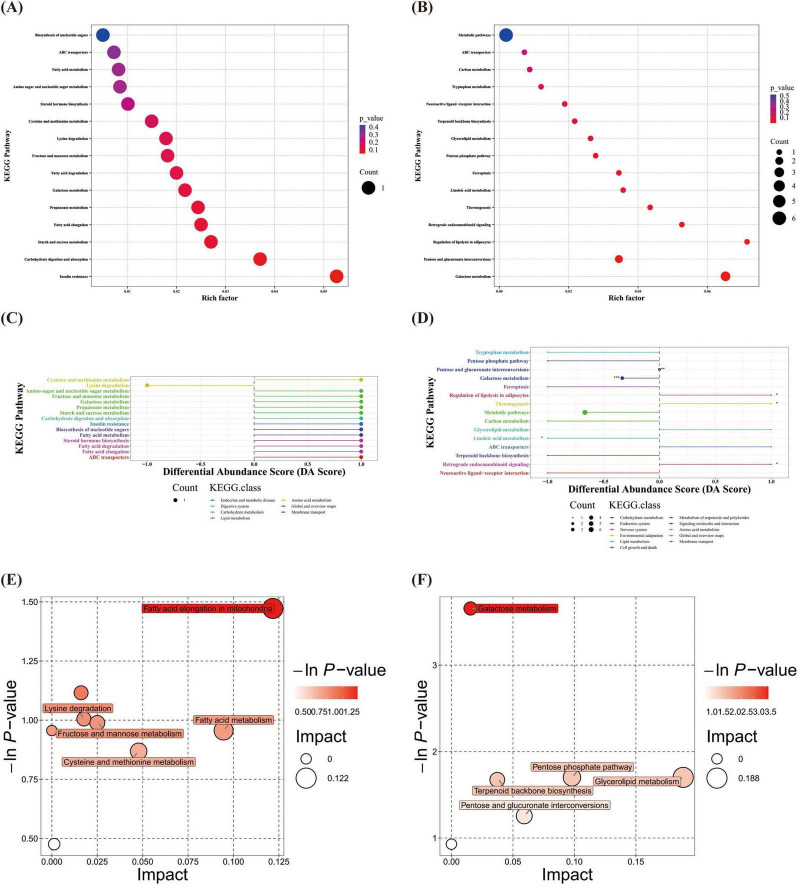
Gut differential metabolites functional prediction at different times. **(A)** KEGG enrichment of gut differential metabolites at T1. **(B)** KEGG enrichment of gut differential metabolites at T2. **(C)** DA score of gut differential metabolites at T1. **(D)** DA score of gut differential metabolites at T2. **(E)** Pathway analysis of gut differential metabolites at T1. **(F)** Pathway analysis of gut differential metabolites at T2. T1, the 10th day of life; T2, the 20th day of life. *< 0.05, **< 0.01, ***< 0.001.

KEGG pathway enrichment analysis revealed no statistically significant pathways between the two groups at T1. At T2, significant differences between the two groups were observed in six pathways. The pathways “regulation of lipolysis in adipocytes,” “thermogenesis” and “retrograde endocannabinoid signaling” were upregulated in the OPT-MOM group, whereas “linoleic acid metabolism” and “galactose metabolism” were downregulated in the OPT-MOM group (Figures 7A–D). An integrated pathway analysis, including enrichment analysis and topological analysis, was performed to identify the key pathways underlying the observed metabolic differences. Our results showed that fatty acid elongation in mitochondria was the most prominent pathway at T1, whereas galactose metabolism was the most prominent pathway at T2 (Figures 7E, F).

### 3.5 The relationship between oral microbiota and gut microbiota

**FIGURE 8:**
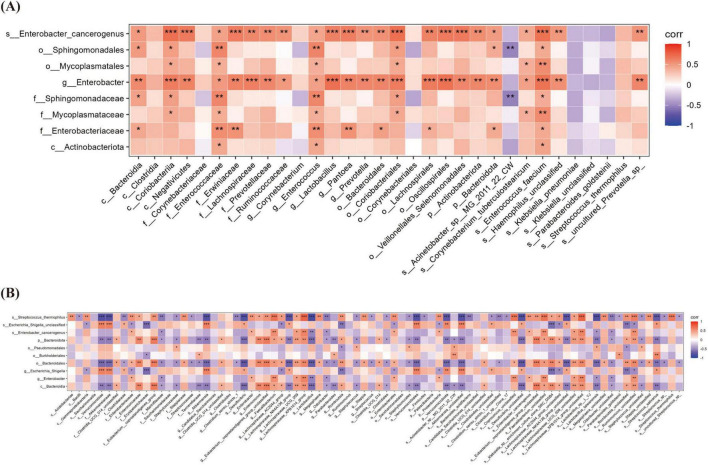
The Relationship between oral microbiota and gut microbiota at different times. **(A)** The correlation heatmap between oral microbiota and gut microbiota at T1. **(B)** The correlation heatmap between oral microbiota and gut microbiota at T2. T1, the 10th day of life; T2, the 20th day of life. *< 0.05, **< 0.01, ***< 0.001.

Significant overlap was observed between the top three most abundant oral and gut microbial taxa at both T0 and T1 (Tables 3, 4). Spearman correlation analysis was performed to investigate the association between oral and gut microbiota. At both T1 and T2, the relative abundance of *Enterobacter* genus and *Enterobacter cancerogenus* in the oropharynx of the OPT-MOM group was significantly lower than that of the control group (Figure 3C). At T1, these oral taxa showed significant positive correlations with Enterococcus faecium, Enterococcaceae family and Enterococcus genus among the intestinal differential bacteria. At T2, they were significantly positively correlated with Enterobacterales order, Enterobacteriaceae family and Proteobacteria phylum in the gut (Figures 8A, B).

**TABLE 3:** The top three most abundant phylum in oral and gut microbiomes across the three periods.

Periods	Group	Oral	Gut
T0	Control	Proteobacteria	Proteobacteria
		Firmicutes	Firmicutes
		Actinobacteria	Actinobacteria
	OPT-MOM	Proteobacteria	Proteobacteria
		Firmicutes	Firmicutes
		Actinobacteria	Bacteroidota
T1	Control	Firmicutes	Firmicutes
		Proteobacteria	Proteobacteria
		Actinobacteria	Actinobacteria
	OPT-MOM	Firmicutes	Firmicutes
		Proteobacteria	Proteobacteria
		Actinobacteria	Actinobacteria
T2	Control	Firmicutes	Proteobacteria
		Proteobacteria	Firmicutes
		Actinobacteria	Actinobacteria
	OPT-MOM	Firmicutes	Firmicutes
		Proteobacteria	Proteobacteria
		Actinobacteria	Actinobacteria

**TABLE 4:** The top three most abundant genus in oral and gut microbiomes across the three periods.

Periods	Group	Oral	Gut
T0	Control	Brevundimonas	Brevundimonas
		Streptococcus	Ralstonia
		Ureaplasma	Ureaplasma
	OPT-MOM	Brevundimonas	Brevundimonas
		Ureaplasma	Streptococcus
		Streptococcus	Ralstonia
T1	Control	Streptococcus	Streptococcus
		Brevundimonas	Enterococcus
		Staphylococcus	Brevundimonas
	OPT-MOM	Streptococcus	Streptococcus
		Staphylococcus	Brevundimonas
		Brevundimonas	Klebsiella
T2	Control	Streptococcus	Klebsiella
		Klebsiella	Enterobacter
		Rothia	Escherichia-Shigella
	OPT-MOM	Streptococcus	Clostridium sensustricto1
		Enterobacter	Streptococcus
		Staphylococcus	Klebsiella

### 3.6 The relationship between gut microbiota and fecal metabolites

**FIGURE 9:**
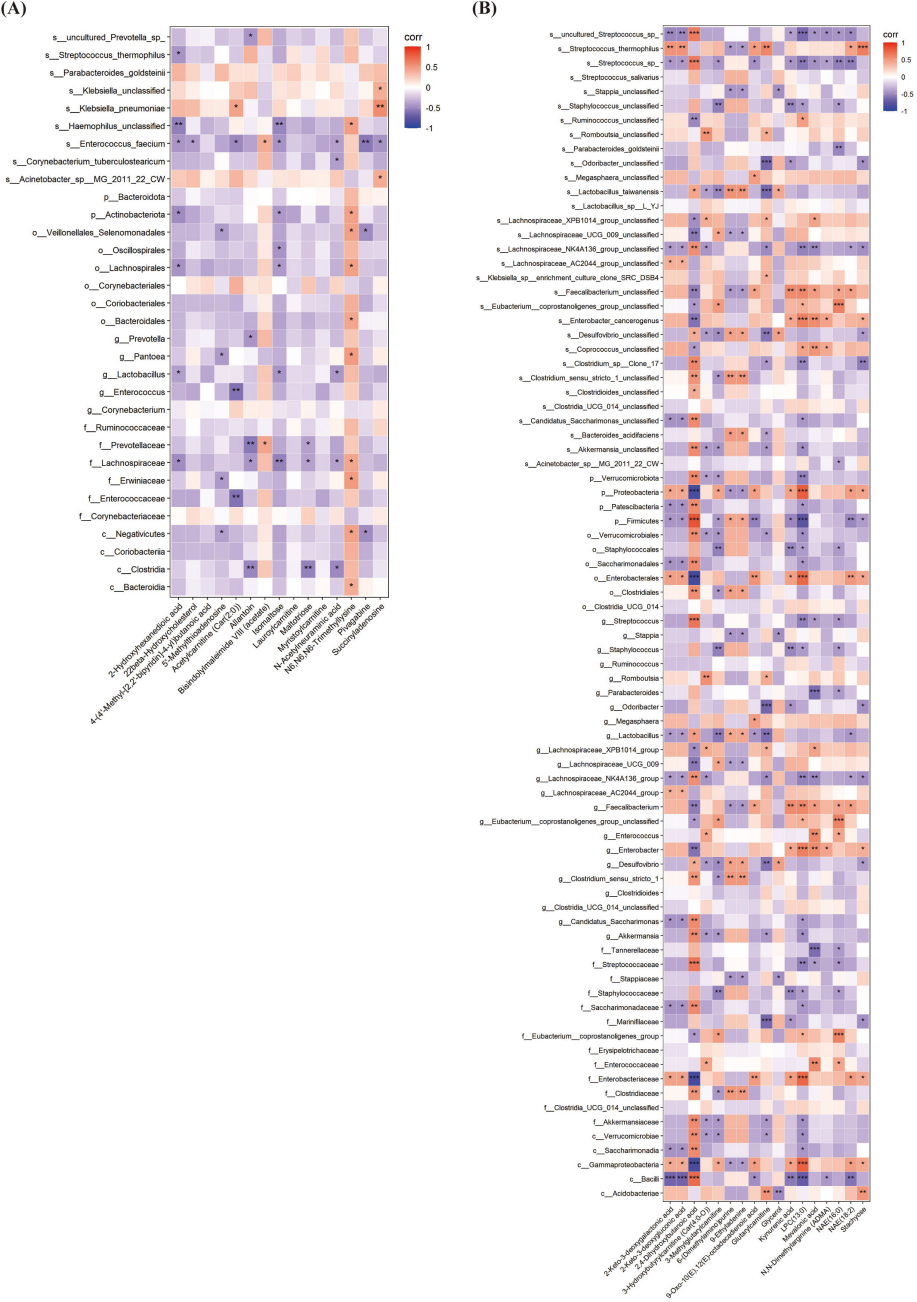
The Relationship between gut microbiota and fecal metabolites at different times. **(A)** The correlation heatmap between gut microbiota and fecal metabolites at T1. **(B)** The correlation heatmap between gut microbiota and fecal metabolites at T2. T1, the 10th day of life; T2, the 20th day of life. *< 0.05, **< 0.01, ***< 0.001.

Correlation analysis between gut differential bacteria and differential metabolites revealed several significant associations. At T1, a strong positive correlation was observed between Klebsiella_pneumoniae and succinyladenosine (r = 0.561, *P* = 0.003), while Enterococcus genus showed a significant negative correlation with acetylcarnitine [Car(2:0)] (r = −0.601, *P* = 0.001). At T2, Firmicutes phylum exhibited a highly positive correlation with 2,4-dihydroxybutanoic acid (r = 0.835, *P* < 0.001), while Enterobacteriaceae family displayed a strong negative correlation with the same metabolite (r = −0.878, *P* < 0.001) (Figures 9A, B).

## 4 Discussion

In this prospective longitudinal pilot study, we investigated the developmental trajectory of oropharyngeal, gut microbiome and metabolomic profiles in VPIs receiving OPT-MOM intervention compared with controls during early life. Additionally, we explored the potential associations between the oropharyngeal and gut microbiomes, as well as the relationships between gut microbial composition and metabolite profiles. The initial colonization of the neonatal microbiota is influenced by various factors, including environment, feeding, and medication. To avoid these confounders, we excluded infants born to mothers with chorioamnionitis and infants on antibiotics for more than 7 days. Our findings provide novel insights into the intricate interactions within the oral-intestinal axis and the complex interplay between the gut microbiome and host immunity in VPIs. These results may offer valuable perspectives on the potential regulatory mechanisms of the OPT-MOM intervention in this vulnerable population, contributing to the development of targeted therapeutic strategies for improving their outcomes.

Very preterm infant pose significant population health burdens due to high mortality, long-term neurodevelopmental impairments, and substantial socioeconomic costs. The etiology of preterm birth is multifactorial and often resulting from the interplay of multiple mechanisms, making it clinically challenging to determine specific causes. In our study cohort, we observed a relatively high prevalence of maternal complications including multiple pregnancies, placental abruption, gestational diabetes mellitus, and gestational hypertension, which may contribute to the occurrence of severe prematurity. Nutritional management constitutes a critical component in the care of very preterm infants. Breastfeeding is widely recognized as beneficial in reducing complications and improving outcomes in this vulnerable population. Previous studies have demonstrated that OAC or OPT-MOM can help increase breastfeeding rates and reduce the incidence of complications in VPIs. In our study, the OPT-MOM group showed lower incidence rates of LOS and NEC compared to the control group. However, these differences did not reach statistical significance, likely due to the limited sample size in our cohort study.

Breast milk harbors a diverse bacterial community and prebiotics which provide pioneer species for the infant’s microbiota and support its development (10, 36). In term newborns, the initial colonizers of the oral cavity are predominantly Staphylococcus and Streptococcus species (37), which are also prevalent as core microbiota in breast milk (38, 39). Currently, there is no consensus regarding the developmental characteristics of the oral microbiota in very preterm infants. Our results showed that the characteristics of the oral flora of very preterm infants at birth were distinct from those of term infants, with the dominant organisms being the Proteobacteria phylum dominated by *Brevundimonas*. Within the first 20 days of life, the oral microbiota underwent significant changes, characterized by a marked decrease in alpha diversity and notable differences in beta diversity, irrespective of OPT-MOM. The Firmicutes phylum, dominated by *streptococcus*, supplanted Proteobacteria phylum as the dominant group in the oral cavity over time, regardless of whether the infants received the OPT-MOM administration. The administration of OPT-MOM demonstrated no statistically significant impact on either alpha or beta diversity of the oral microbiota. These findings are consistent with previous studies (24–27). The analysis revealed distinct microbial compositional differences between two groups. In the OPT-MOM group, a significant reduction was observed in the relative abundance of some potentially pathogenic genera, including *Escherichia-Shigella* and *Enterobacter*. Conversely, the OPT-MOM group demonstrated a marked increase in the relative abundance of *Rothia*, which is known to produce short-chain fatty acids (SCFAs) (40). These SCFAs play a crucial role in regulating the colonization of intestinal microbiota during early life, promoting the maturation of the intestinal mucosal barrier, modulating intestinal immunity and facilitating the development of the immune and nervous systems (41–43). Integrating the results of the bacterial phenotype prediction analyses, our data suggest that the OPT-MOM intervention could potentially promote the establishment of a normal oral microbiota.

The gut of term infants undergoes rapid colonization by bacteria such as Bifidobacterium after birth (44–46). Consistent with previous studies (47), our findings indicate that the gut microbiota of preterm infants is not initially dominated by Bifidobacterium, potentially due to factors including feeding practices, cesarean delivery, antibiotic administration, and mother-infant separation. To the best of our knowledge, the impact of OPT-MOM on gut microbiota remains a relatively underexplored area. The existing literature is limited to a single study that examined the influence of OAC on intestinal microbiota through conventional bacterial culture methods, which demonstrated no statistically significant alterations (48). Our results showed that longitudinal assessment of gut microbiota alpha diversity, measured by three different indices (Shannon, Simpson and Chao1), revealed no statistically significant temporal trends in either group, while beta diversity showed significant temporal differences. Notably, we observed that prolonged OPT-MOM significantly altered the composition of the gut microbiota. While no statistically significant differences in beta diversity were observed between the two groups at birth and 10 days postpartum, a significant difference emerged at 20 days postpartum, indicating a time-dependent effect of OPT-MOM on gut microbial community structure. Our analysis demonstrated that OPT-MOM significantly influenced the temporal dynamics of the dominant gut bacterial phyla. At birth, Proteobacteria was the dominant phylum in both groups. By 20 days postpartum, the Control group continued to exhibit the highest relative abundance of Proteobacteria. In contrast, the OPT-MOM group showed a continuous decrease in the abundance of Proteobacteria, while the abundance of Firmicutes increased, eventually becoming the dominant phylum. This microbial shift observed in the OPT-MOM group represents a potentially beneficial modification for reducing the risk of NEC in preterm infants, as emerging evidence suggests that elevated Proteobacteria abundance coupled with reduced Firmicutes levels are characteristic features of gut microbiota dysbiosis preceding the development of NEC (49). Our study also showed that at 20 days postpartum, the relative abundance of Streptococcus and Staphylococcus genera increased significantly in the OPT-MOM group. These genera are known to be dominant in breast milk (50), suggesting that OPT-MOM may facilitate the transfer of bacteria from breast milk to the neonatal gut. This finding is consistent with previous reports that breast milk can transfer bacteria to the infant’s oral cavity and gut (38, 50). Bacterial phenotype prediction analysis revealed a significant reduction in the potential pathogenicity of the gut microbiota in the OPT-MOM group compared to the control group at 20 days after birth. This substantial reduction in microbial pathogenicity potential suggests that the protective mechanism of OPT-MOM in very preterm infants may be mediated by the establishment of a more balanced and commensal-dominated gut microbiota.

Emerging evidence from adult studies has established a significant link between the oral and gut microbiota through the oral-gut axis, which plays a critical role in the pathogenesis of various systemic diseases, including hypertension and inflammatory bowel disease (51–53). However, the specific associations between oral and gut microbiota in preterm infants and their impact on preterm infants remain poorly understood. In this present study, similar dominant phylum and genera were identified between the oral cavity and the gut of preterm infants at the first day and the 10th day of life. This finding indicates a potential correlation or synchronization between the evolutionary trajectories of oral and intestinal microbiota.

The metabolome comprises thousands of small molecular metabolites, most of which are products of co-metabolism between the microbiota and the host, and play an important role in maintaining host homeostasis. Due to the limited amount of saliva samples, which may be insufficient for analysis, this study primarily focuses on intestinal metabolites. Accumulating evidence suggests that fecal metabolomics serves as a valuable indicator of the complex interactions among the host, dietary factors, and gut microbiota (54). However, the developmental trajectory of the gut metabolome in preterm infants during early life remains poorly characterized. Our results showed that with increasing postnatal age, the intestinal metabolic profiles of both groups underwent significant changes. Following the OPT-MOM intervention, the structure of intestinal metabolites in the OPT-MOM group was significantly different from that in the control group. Metabolomic analysis revealed significantly elevated levels of several key metabolites in the OPT-MOM intervention group compared to controls, including N-acetylneuraminic acid, myristoylcarnitine, lauroylcarnitine, acetylcarnitine [Car(2:0)] and 2,4-dihydroxybutanoic acid. N-acetylneuraminic acid is a key component of human milk oligosaccharides (HMOs) (55), which play a vital role in the growth and differentiation of neural cells (56, 57). Studies have shown that N-acetylneuraminic acid can improve cognitive ability and memory in rats (58). Myristoylcarnitine, lauroylcarnitine and acetylcarnitine are acylated derivatives of L-carnitine, of which acetylcarnitine [Car(2:0)] is a short-chain acylcarnitine. Higher levels of L-carnitine and short-chain acylcarnitines are positively correlated with rapid growth within the first year of life (55). 2,4-Dihydroxybutanoic acid, a hydroxylated derivative of butyric acid, shares structural similarities with butyrate, a key member of short-chain fatty acids (SCFAs) known for its significant physiological roles. Butyrate exhibits potent anti-inflammatory properties, effectively suppressing the expression of pro-inflammatory cytokines, including IL-1β, IL-6, and TNF-α, which are induced by lipopolysaccharide (LPS) in both pulmonary and intestinal tissues (59, 60). Furthermore, butyrate has been demonstrated to inhibit microglial activation in the cerebral cortex and hippocampus, thereby attenuating neuroinflammation induced by NEC (61). Therefore, the implementation of OPT-MOM may potentially increase the concentration of specific key metabolites that may play a pivotal role in modulating health outcomes and reducing disease risk in preterm infants.

Gut metabolites can be significantly shaped by the gut microbiome and serve as key mediators of host-microbiota interactions, which requires further mechanistic studies. The gut microbiota can regulate signaling pathways that maintain intestinal mucosal homeostasis and modulate intestinal immunity through the production of specific metabolites (62). Bioinformatic analysis revealed that the protective effects of OPT-MOM may be mediated through specific metabolic pathways, particularly mitochondrial fatty acid elongation and galactose metabolism, which emerged as the most significantly associated pathways. Furthermore, microbial-metabolite correlation analysis revealed a positive association between Klebsiella pneumoniae and succinyladenosine, while *Enterococcus* showed a negative correlation with acetylcarnitine. Notably, Firmicutes showed a strong positive correlation with 2,4-dihydroxybutanoic acid, in contrast to the negative correlation observed for *Enterobacteriaceae*. These findings provide valuable insights into potential mechanisms underlying the biological effects of OPT-MOM and suggest promising directions for future mechanistic investigations.

The primary limitation of this study is the relatively small sample size. We acknowledge that the current sample size may limit the generalizability of our findings and the statistical power of the analyzes. However, this preliminary investigation can serve as a crucial step in hypothesis generation and provide valuable insights into the feasibility of conducting larger trials in the future. Additionally, this study was technically limited by the inherent constraints of the 16S rRNA gene sequencing methodology. Specifically, this approach is restricted by its relatively limited sequencing depth and inability to provide comprehensive quantitative functional profiling of microbial communities. Future investigations will incorporate expanded cohort validation combined with integrated targeted metabolomics and shotgun metagenomic sequencing to elucidate the biological pathways and molecular interactions implicated by our findings. Complementary gnotobiotic animal studies will be conducted to establish causal relationships and assess functional mechanisms.

## 5 Conclusion

This study demonstrated that the administration of OPT-MOM promoted the establishment of favorable microbial communities in both oral and intestinal ecosystems of preterm infants, potentially facilitating the biosynthesis of health-promoting metabolites that play vital roles in early-life development. These findings provide novel insights into the protective role of OPT-MOM in preterm infants, and may improve our understanding of the intricate crosstalk between gut microbial colonization and host immune system development during early life. Further large-scale studies are warranted to validate these preliminary findings, complemented by comprehensive mechanistic investigations to elucidate the underlying biological pathways and molecular interactions.

## Data Availability

The datasets presented in this study can be found in the National Center for Biotechnology Information (NCBI) under BioProject ID PRJNA1274605.

## References

[1] Kaffe K Syrogiannopoulos G Petinaki E Goudesidou M Kalaitzi A Gounaris A Epidemiology and outcomes of late-onset neonatal sepsis in preterm infants in a tertiary hospital. *Children.* (2025) 12:532. 10.3390/children12050532 40426711 PMC12110585

[2] Ofek Shlomai N Tayeb M Abu Omar R Eventov Friedman S. Changes in the incidence and severity of NEC over the last decade: a single-center study. *JCM.* (2025) 14:3551. 10.3390/jcm14103551 40429546 PMC12112256

[3] Drommelschmidt K Mayrhofer T Müller H Foldyna B Raudzus J Göricke S CMRI-detected brain injuries and clinical key risk factors associated with adverse neurodevelopmental outcomes in very preterm infants. *Sci Rep.* (2025) 15:18221. 10.1038/s41598-025-02539-1 40415084 PMC12104413

[4] Alrahili M Halabi S Al Essa A Alrsheedi S Almuqati R Althubaiti M Delayed admission temperature normalisation in preterm infants <32 weeks: impact on mortality and neonatal morbidities. *bmjpo.* (2025) 9:e003473. 10.1136/bmjpo-2025-003473 40404190 PMC12097038

[5] Miller J Tonkin E Damarell R McPhee A Suganuma M Suganuma H A systematic review and meta-analysis of human milk feeding and morbidity in very low birth weight infants. *Nutrients.* (2018) 10:707. 10.3390/nu10060707 29857555 PMC6024377

[6] Joosten K Vermeulen M. Principles of feeding the preterm infant. *Clin Nutr ESPEN.* (2024) 59:320–7. 10.1016/j.clnesp.2023.12.016 38220393

[7] Quigley M Embleton N McGuire W. Formula versus donor breast milk for feeding preterm or low birth weight infants. *Cochrane Database Syst Rev.* (2019) 7:CD002971. 10.1002/14651858.CD002971.pub5 31322731 PMC6640412

[8] Muts J Van Keulen B Van Goudoever J Van Den Akker C. Formula protein versus human milk protein and the effects on growth in preterm born infants. *Curr Opin Clin Nutr Metab Care.* (2025) 28:33–8. 10.1097/MCO.0000000000001084 39659212 PMC11634171

[9] Ghith A Maleki R Grzeskowiak L Amir L Ingman W. Challenges and opportunities in quantifying bioactive compounds in human breastmilk. *Biomolecules.* (2025) 15:325. 10.3390/biom15030325 40149861 PMC11940641

[10] Natal A de Paula Menezes R de Brito Röder D. Role of maternal milk in providing a healthy intestinal microbiome for the preterm neonate. *Pediatr Res.* (2024): 10.1038/s41390-024-03751-x 39663425

[11] Kim S Yi D. Components of human breast milk: from macronutrient to microbiome and microRNA. *Clin Exp Pediatr.* (2020) 63:301–9. 10.3345/cep.2020.00059 32252145 PMC7402982

[12] Socha-Banasiak A Pawłowska M Czkwianianc E Pierzynowska K. From intrauterine to extrauterine life—the role of endogenous and exogenous factors in the regulation of the intestinal microbiota community and gut maturation in early life. *Front Nutr.* (2021) 8:696966. 10.3389/fnut.2021.696966 34977104 PMC8718557

[13] OuYang X Yang C Xiu W Hu Y Mei S Lin Q. Oropharyngeal administration of colostrum for preventing necrotizing enterocolitis and late-onset sepsis in preterm infants with gestational age ≤ 32 weeks: a pilot single-center randomized controlled trial. *Int Breastfeed J.* (2021) 16:59. 10.1186/s13006-021-00408-x 34419090 PMC8379587

[14] Rodriguez N Meier P Groer M Zeller J. Oropharyngeal administration of colostrum to extremely low birth weight infants: theoretical perspectives. *J Perinatol.* (2009) 29:1–7. 10.1038/jp.2008.130 18769379 PMC2730520

[15] Huo M Liu C Mei H Zhang Y Liu C Song D Intervention effect of oropharyngeal administration of colostrum in preterm infants: a meta-analysis. *Front Pediatr.* (2022) 10:895375. 10.3389/fped.2022.895375 35832583 PMC9271762

[16] Fu Z Huang C Lei L Chen L Wei L Zhou J The effect of oropharyngeal colostrum administration on the clinical outcomes of premature infants: a meta-analysis. *Int J Nurs Stud.* (2023) 144:104527. 10.1016/j.ijnurstu.2023.104527 37295286

[17] Anne R Kumar J Kumar P Meena J. Effect of oropharyngeal colostrum therapy on neonatal sepsis in preterm neonates: a systematic review and meta-analysis. *J Pediatr Gastroenterol Nutr.* (2024) 78:471–87. 10.1002/jpn3.12085 38314925

[18] Kumar J Meena J Ranjan A Kumar P. Oropharyngeal application of colostrum or mother’s own milk in preterm infants: a systematic review and meta-analysis. *Nutr Rev.* (2023) 81:1254–66. 10.1093/nutrit/nuad002 36718589

[19] Gates A Hair A Salas A Thompson A Stansfield B. Nutrient composition of donor human milk and comparisons to preterm human milk. *J Nutr.* (2023) 153:2622–30. 10.1016/j.tjnut.2023.07.012 37517552

[20] Gialeli G Kapetanaki A Panagopoulou O Vourna P Michos A Kanaka-Gantenbein C Supplementation of mother’s own milk with preterm donor human milk: impact on protein intake and growth in very low birth weight infants—a randomized controlled study. *Nutrients.* (2023) 15:566. 10.3390/nu15030566 36771273 PMC9919101

[21] Underwood M. Human milk for the premature infant. *Pediatric Clin North Am.* (2013) 60:189–207. 10.1016/j.pcl.2012.09.008 23178065 PMC3508468

[22] Walker A. Breast milk as the gold standard for protective nutrients. *J Pediatr.* (2010) 156:S3–7. 10.1016/j.jpeds.2009.11.021 20105662

[23] Sudeep K Kumar J Ray S Dutta S Aggarwal R Kumar P. Oral application of colostrum and mother’s own milk in preterm infants—A randomized. *Controlled Trial. Indian J Pediatr.* (2022) 89:579–86. 10.1007/s12098-021-03982-4 35006497

[24] Sohn K Kalanetra K Mills D Underwood M. Buccal administration of human colostrum: impact on the oral microbiota of premature infants. *J Perinatol.* (2016) 36:106–11. 10.1038/jp.2015.157 26658119

[25] Romano-Keeler J Azcarate-Peril M Weitkamp J Slaughter J McDonald W Meng S Oral colostrum priming shortens hospitalization without changing the immune-microbial milieu. *J Perinatol.* (2017) 37:36–41. 10.1038/jp.2016.161 27684425 PMC5215726

[26] Cortez R Fernandes A Sparvoli L Padilha M Feferbaum R Neto C Impact of oropharyngeal administration of colostrum in preterm newborns’. *Oral Microbiome. Nutrients.* (2021) 13:4224. 10.3390/nu13124224 34959775 PMC8703686

[27] Thatrimontrichai A Surachat K Singkhamanan K Thongsuksai P. Long duration of oral care using mother’s own milk influences oral microbiota and clinical outcomes in very-low-birthweight infants: randomized controlled trial. *Pediatr Infect Dis J.* (2023) 42:804–10. 10.1097/INF.0000000000004002 37343216

[28] Phillips-Farfán B Gómez-Chávez F Medina-Torres E Vargas-Villavicencio J Carvajal-Aguilera K Camacho L. Microbiota signals during the neonatal period forge life-long immune responses. *IJMS.* (2021) 22:8162. 10.3390/ijms22158162 34360926 PMC8348731

[29] Ebm Group, Neonatologist Society, Chinese Medical Doctor Association. [Clinical guidelines for the diagnosis and treatment of feeding intolerance in preterm infants (2020)]. *Zhongguo Dang Dai Er Ke Za Zhi.* (2020) 22:1047–55. 10.7499/j.issn.1008-8830.2008132 33059799 PMC7568993

[30] Bell M Ternberg J Feigin R Keating J Marshall R Barton L Neonatal necrotizing enterocolitis. Therapeutic decisions based upon clinical staging. *Ann Surg.* (1978) 187:1–7. 10.1097/00000658-197801000-00001 413500 PMC1396409

[31] Walsh MC Kliegman RM. Necrotizing enterocolitis: treatment based on staging criteria. *Pediatr Clin North Am.* (1986) 33:179–201. 10.1016/S0031-3955(16)34975-6 3081865 PMC7131118

[32] Dong Y Speer C. Late-onset neonatal sepsis: recent developments. *Arch Dis Child Fetal Neonatal Ed.* (2015) 100:F257–63. 10.1136/archdischild-2014-306213 25425653 PMC4413803

[33] Fierson W American Academy of Pediatrics Section on Ophthalmology, American Academy of Ophthalmology, American Association for Pediatric Ophthalmology and Strabismus, American Association of Certified Orthoptists, Chiang MF Screening examination of premature infants for retinopathy of prematurity. *Pediatrics.* (2018) 142:e20183061. 10.1542/peds.2018-3061 30478242

[34] Voynow J. “New” bronchopulmonary dysplasia and chronic lung disease. *Paediatr Respiratory Rev.* (2017) 24:17–8. 10.1016/j.prrv.2017.06.006 28697967

[35] Maller V Cohen H. Neurosonography: assessing the premature infant. *Pediatr Radiol.* (2017) 47:1031–45. 10.1007/s00247-017-3884-z 28779189

[36] Selma-Royo M Calvo-Lerma J Bäuerl C Esteban-Torres M Cabrera-Rubio R Collado M. Human milk microbiota: what did we learn in the last 20 years? *Microbiome Res Rep.* (2022) 1:19. 10.20517/mrr.2022.05 38046359 PMC10688795

[37] Sampaio-Maia B Monteiro-Silva F. Acquisition and maturation of oral microbiome throughout childhood: an update. *Dental Res J.* (2014) 11:291–301. PMC4119360 25097637

[38] Ruiz L Bacigalupe R García-Carral C Boix-Amoros A Argüello H Silva C Microbiota of human precolostrum and its potential role as a source of bacteria to the infant mouth. *Sci Rep.* (2019) 9:8435. 10.1038/s41598-019-42514-1 31182726 PMC6557856

[39] Notarbartolo V Giuffrè M Montante C Corsello G Carta M. Composition of human breast milk microbiota and its role in children’s health. *Pediatr Gastroenterol Hepatol Nutr.* (2022) 25:194–210. 10.5223/pghn.2022.25.3.194 35611376 PMC9110848

[40] Owens J Qiu H Knoblich C Gerjevic L Izard J Xu L Feeding intolerance after pediatric cardiac surgery is associated with dysbiosis, barrier dysfunction, and reduced short-chain fatty acids. *Am J Physiol Gastrointestinal Liver Physiol.* (2024) 327:G685–96. 10.1152/ajpgi.00151.2024 39224072 PMC11559637

[41] Alsharairi N. Therapeutic potential of gut microbiota and its metabolite short-chain fatty acids in neonatal necrotizing enterocolitis. *Life.* (2023) 13:561. 10.3390/life13020561 36836917 PMC9959300

[42] Mann E Lam Y Uhlig H. Short-chain fatty acids: linking diet, the microbiome and immunity. *Nat Rev Immunol.* (2024) 24:577–95. 10.1038/s41577-024-01014-8 38565643

[43] Li L Yang J Liu T Shi Y. Role of the gut-microbiota-metabolite-brain axis in the pathogenesis of preterm brain injury. *Biomed Pharmacother.* (2023) 165:115243. 10.1016/j.biopha.2023.115243 37517290

[44] Brink L Mercer K Piccolo B Chintapalli S Elolimy A Bowlin A Neonatal diet alters fecal microbiota and metabolome profiles at different ages in infants fed breast milk or formula. *Am J Clin Nutr.* (2020) 111:1190–202. 10.1093/ajcn/nqaa076 32330237 PMC7266684

[45] Kok C Brabec B Chichlowski M Harris C Moore N Wampler J Stool microbiome, pH and short/branched chain fatty acids in infants receiving extensively hydrolyzed formula, amino acid formula, or human milk through two months of age. *BMC Microbiol.* (2020) 20:337. 10.1186/s12866-020-01991-5 33167908 PMC7650147

[46] Li N Yan F Wang N Song Y Yue Y Guan J Distinct gut microbiota and metabolite profiles induced by different feeding methods in healthy chinese infants. *Front Microbiol.* (2020) 11:714. 10.3389/fmicb.2020.00714 32435235 PMC7219020

[47] Rozé J Ancel P Marchand-Martin L Rousseau C Montassier E Monot C Assessment of neonatal intensive care unit practices and preterm newborn gut microbiota and 2-year neurodevelopmental outcomes. *JAMA Netw Open.* (2020) 3:e2018119. 10.1001/jamanetworkopen.2020.18119 32965499 PMC7512059

[48] Wang H Li Q Xu X Hu X. Effects of sublingual colostrum application on oral and intestinal flora of extremely low birth weight infants. *Endocr Metab Immune Disord Drug Targets.* (2024) 24:489–94. 10.2174/1871530323666230913105820 37711000

[49] Moschino L Verlato G Duci M Cavicchiolo M Guiducci S Stocchero M The metabolome and the gut microbiota for the prediction of necrotizing enterocolitis and spontaneous intestinal perforation: a systematic review. *Nutrients.* (2022) 14:3859. 10.3390/nu14183859 36145235 PMC9506026

[50] Fehr K Moossavi S Sbihi H Boutin R Bode L Robertson B Breastmilk feeding practices are associated with the co-occurrence of bacteria in mothers’ milk and the infant gut: the child cohort study. *Cell Host Microbe.* (2020) 28:9. 10.1016/j.chom.2020.06.009 32652062

[51] Kunath B De Rudder C Laczny C Letellier E Wilmes P. The oral–gut microbiome axis in health and disease. *Nat Rev Microbiol.* (2024) 22:791–805. 10.1038/s41579-024-01075-5 39039286

[52] Chen B Lin W Li Y Bi C Du L Liu Y Roles of oral microbiota and oral-gut microbial transmission in hypertension. *J Adv Res.* (2023) 43:147–61. 10.1016/j.jare.2022.03.007 36585105 PMC9811375

[53] Abdelbary M Hatting M Bott A Dahlhausen A Keller D Trautwein C The oral-gut axis: salivary and fecal microbiome dysbiosis in patients with inflammatory bowel disease. *Front Cell Infect Microbiol.* (2022) 12:1010853. 10.3389/fcimb.2022.1010853 36275026 PMC9585322

[54] Koh A Bäckhed F. From association to causality: the role of the gut microbiota and its functional products on host metabolism. *Molecular Cell.* (2020) 78:584–96. 10.1016/j.molcel.2020.03.005 32234490

[55] Ouyang R Ding J Huang Y Zheng F Zheng S Ye Y Maturation of the gut metabolome during the first year of life in humans. *Gut Microbes.* (2023) 15:2231596. 10.1080/19490976.2023.2231596 37424334 PMC10334852

[56] Rajhans P Mainardi F Austin S Sprenger N Deoni S Hauser J The Role of human milk oligosaccharides in myelination, socio-emotional and language development: observational data from breast-fed infants in the United States of America. *Nutrients.* (2023) 15:4624. 10.3390/nu15214624 37960278 PMC10649431

[57] Liu F Van Der Molen J Kuipers F Van Leeuwen S. Quantitation of bioactive components in infant formulas: milk oligosaccharides, sialic acids and corticosteroids. *Food Res Int.* (2023) 174:113589. 10.1016/j.foodres.2023.113589 37986455

[58] Silveira R Corso A Procianoy R. The influence of early nutrition on neurodevelopmental outcomes in preterm infants. *Nutrients.* (2023) 15:4644. 10.3390/nu15214644 37960297 PMC10648100

[59] Liu J Chang G Huang J Wang Y Ma N Roy A Sodium butyrate inhibits the inflammation of lipopolysaccharide-induced acute lung injury in mice by regulating the toll-like receptor 4/nuclear factor κB signaling pathway. *J Agric Food Chem.* (2019) 67:1674–82. 10.1021/acs.jafc.8b06359 30661349

[60] Sun Q Ji Y Wang Z She X He Y Ai Q Sodium butyrate alleviates intestinal inflammation in mice with necrotizing enterocolitis. *Mediators Inflammation.* (2021) 2021:6259381–6259312. 10.1155/2021/6259381 34675753 PMC8526205

[61] Martinez M Yu W Menden H Lei T Monaghan-Nichols P Sampath V. Butyrate suppresses experimental necrotizing enterocolitis–induced brain injury in mice. *Front Pediatr.* (2023) 11:1284085. 10.3389/fped.2023.1284085 38130941 PMC10733464

[62] McCallum G Tropini C. The gut microbiota and its biogeography. *Nat Rev Microbiol.* (2024) 22:105–18. 10.1038/s41579-023-00969-0 37740073

