# Supplementary material for: Influence of oropharyngeal therapy with mother’s own milk on the microbiome and metabolome of very preterm infants: a pilot study
Source: Front Nutr. 2025 Aug 1;12:1647379. doi: 10.3389/fnut.2025.1647379 (PMC12355658; doi:10.3389/fnut.2025.1647379)
Supplement: Supplementary file 1 [file Image_1.pdf]

# *Supplementary Material*

## Supplementary Figures

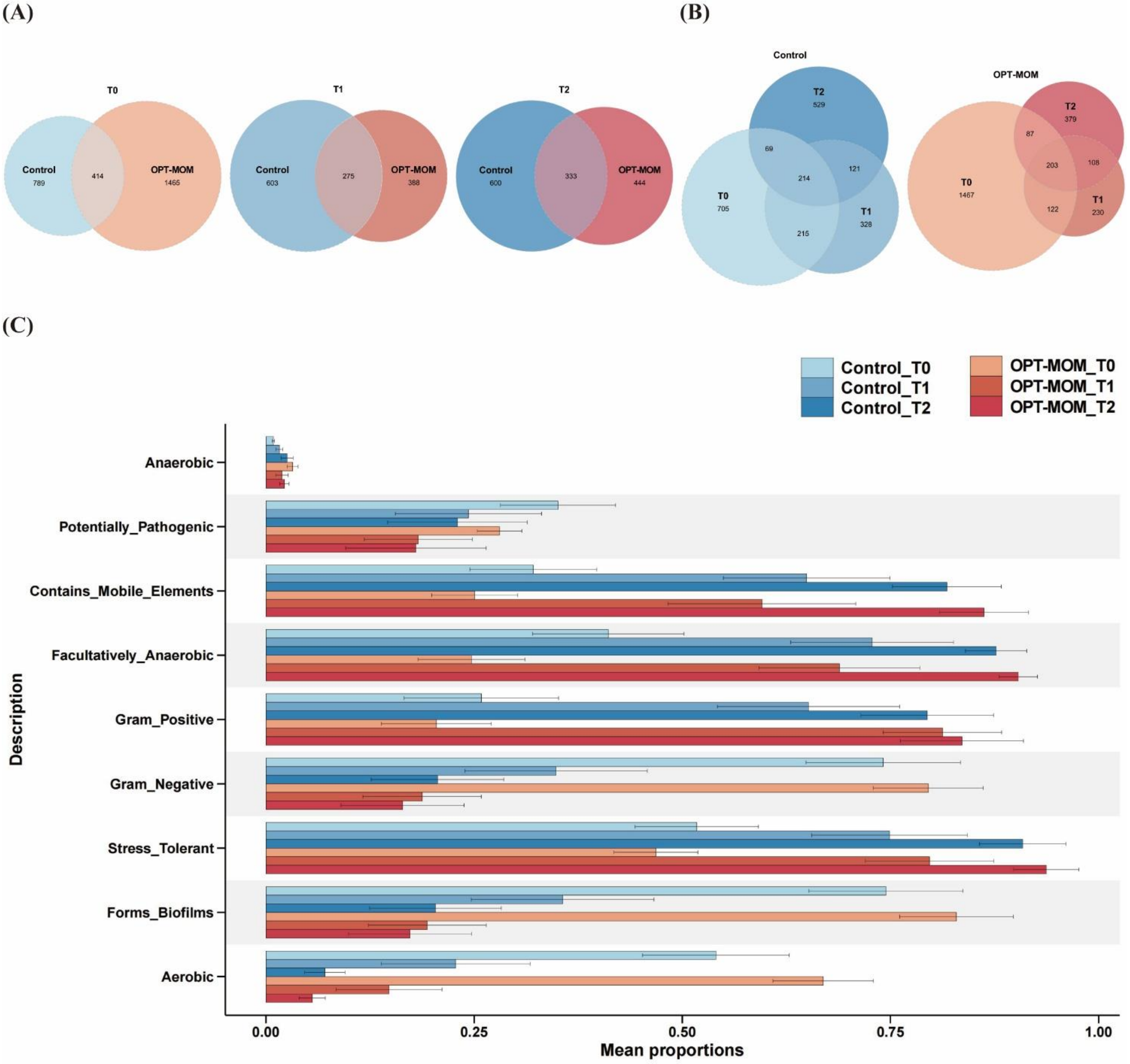


**Supplemental Figure 1.** Comparison of oral microbiota. (A) ASVs comparison between the two groups at different times. (B) ASVs of control group and OPT-MOM group over time. (C) BugBase potential prediction for phenotypic functions of bacteria of two groups at different times. Control, control group; OPT-MOM, oropharyngeal therapy with mother's own milk group. T0, the first day of life; T1, the 10th day of life; T2, the 20th day of life.

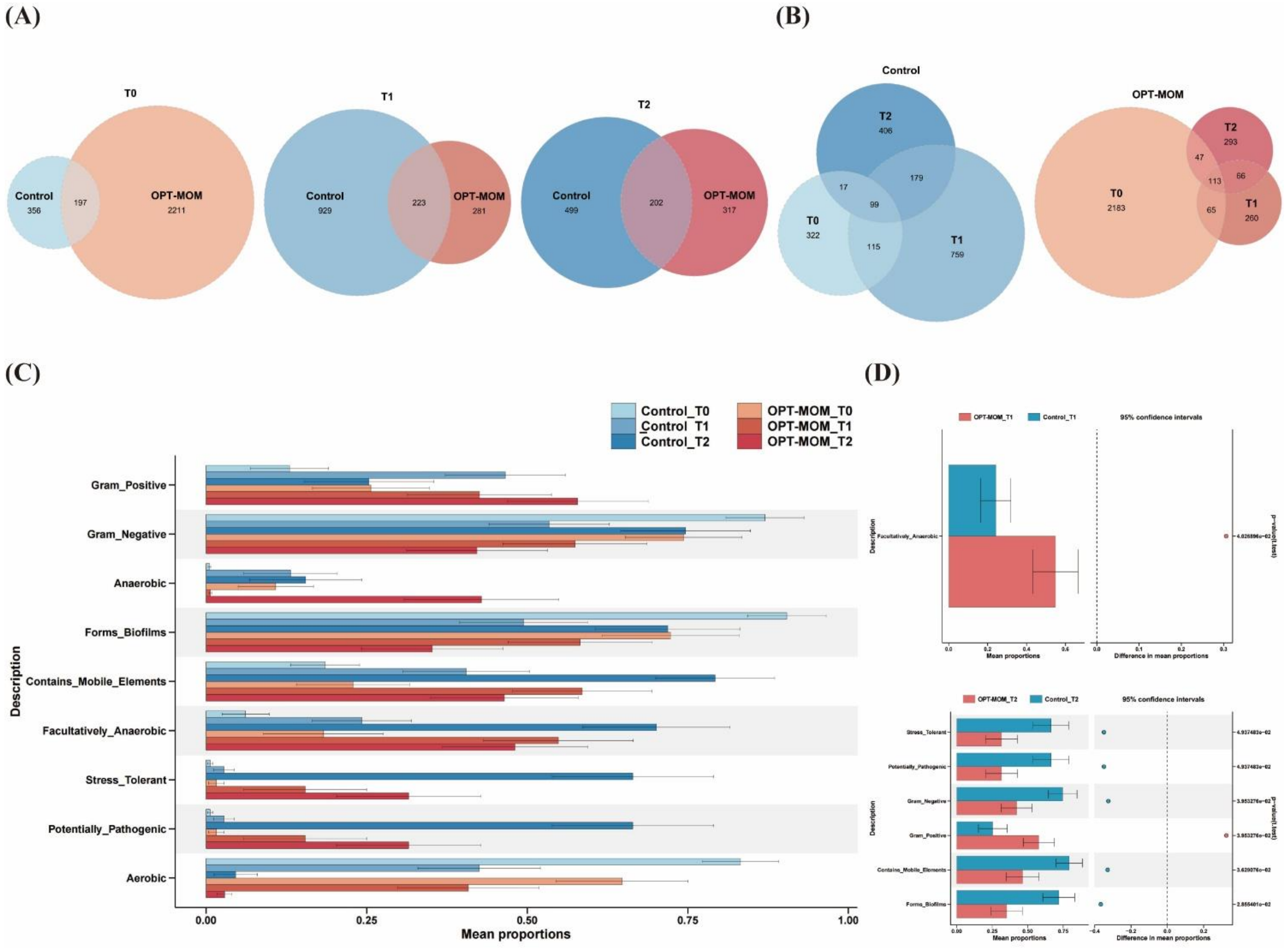


**Supplemental Figure 2.** Comparison of gut microbiota. (A) ASVs comparison between the two groups at different times. (B) ASVs of control group and OPT-MOM group over time. (C) BugBase potential prediction for phenotypic functions of bacteria of two groups at different times. (D) Statistically different results of phenotypic prediction of bacteria comparison. Control, control group; OPT-MOM, oropharyngeal therapy with mother's own milk group. T0, the first day of life; T1, the 10th day of life; T2, the 20th day of life.

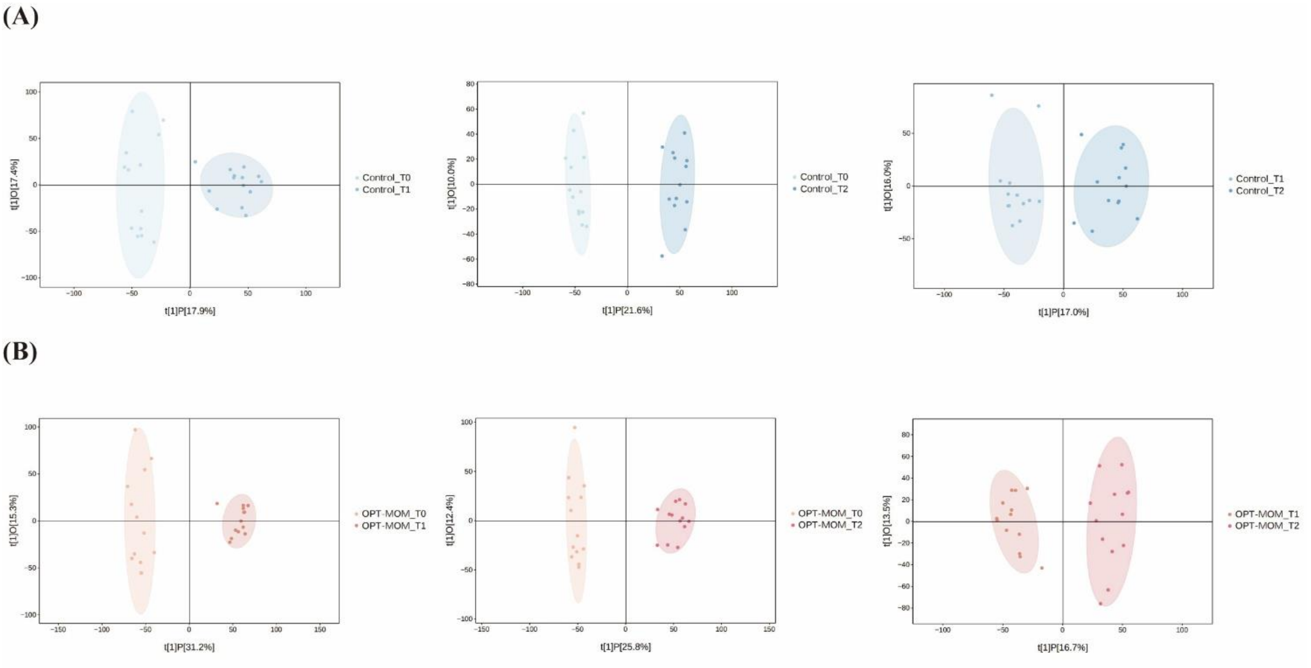


**Supplemental Figure 3.** Comparison of gut metabolite. (A) OPLS-DA analysis of control group over time. (B) OPLS-DA analysis of OPT-MOM group over time. Control, control group; OPT-MOM, oropharyngeal therapy with mother's own milk group. T0, the first day of life; T1, the 10th day of life; T2, the 20th day of life.
